# The autonomy of sport concept: a scoping review

**DOI:** 10.3389/fspor.2025.1593673

**Published:** 2025-06-19

**Authors:** Viktoriia Smirnova, Pedro José Mercado Jaén, Holger Preuss, Thomas Könecke, Mathias Schubert

**Affiliations:** ^1^ Institute of Sport Science, Department of Sport Economics, Sociology and History, Johannes Gutenberg-Universität Mainz, Mainz, Germany; ^2^ Department of Law, European University Institute, Florence, Italy; ^3^ Physical Activity, Sports & Health Research Group, KU Leuven, Leuven, Belgium; ^4^ iCERIS (interdisciplinary Centre for Ethics, Regulation and Integrity in Sport), KU Leuven, Leuven, Belgium; ^5^ LISS (KU Leuven Institute of Sports Science), KU Leuven, Leuven, Belgium

**Keywords:** governance, regulators, Olympic movement, self-regulation, governance evolution

## Abstract

The autonomy of sport concept can be considered a fundamental principle within international sport governance. In essence, the principle signifies the right to self-regulation and reflects the ability of sport governing bodies (SGBs) to determine their own structures and rules, free from interference by external actors. Despite growing academic and practitioner interest, there is still no consensus as to what the term “autonomy” exactly means and how widely the principle is (supposed to be) applied in a changing world of sport. This article systematically maps the extent of research on the concept of sport autonomy, including its applications and limitations. Based on the PRISMA Extension for Scoping Reviews (PRISMA-ScR), our review identified 205 records examining sport autonomy between 1982 and 2024. The results reveal a notable increase in sport autonomy research over the last decade, demonstrating the increasing importance of the topic. The majority of records is non-empirical and focus on international multi-sport governing bodies, highlighting a Western-dominated nature on sport autonomy. Based on a qualitative content analysis, we contribute to theory by extending the multidimensional understanding of sport autonomy. Autonomy is a dynamic and multifaceted concept that needs to be studied in the dimensions of the interlinked autonomies. Our findings indicate that the borders between autonomies in sport governance practice are ambiguous, calling for more empirically driven research in future assessments. The great heterogeneity of SGBs requires a sophisticated deconstruction of different dimensions and conceptualisations of autonomy of sport, focusing on autonomy as a spectrum.

## 1 Introduction

The autonomy of sport has, arguably, become one of the most controversial concepts of the Olympic Movement (1). It has been considered an integral part of global sport governance since the International Olympic Committee (IOC) adopted the doctrine of sport autonomy in the mid-20th century (2). Making recognition of the autonomy at that time was a way of resisting government pressures predominantly from the countries of the Soviet bloc, which were beginning to join the Olympic Movement (3). Ever since, every new edition to the Olympic Charter codifies the rights and obligations of autonomy as a fundamental principle of Olympism (4). This principle signifies the right to self-regulation and reflects the ability of sport governing bodies (SGBs) to determine their own structures, governance and sporting rules, free from interference by governments and other external actors (5).

The principle of the autonomy of sport is not only a long-standing tradition but is also recognised as a “universal fundamental ethical principle” of the Olympic Movement, enshrined in the IOC's Code of Ethics (4). Furthermore, the Olympic Charter states autonomy of sport as the fifth “Fundamental Principle of Olympism” (6). The strategic role of the autonomy of sport for the Olympic Movement is also reflected in the Olympic Charter, which states that one of the missions of the IOC is “to preserve the autonomy of sport” (4). As one of the working principles of the Olympic Movement, the aim of autonomy of sport is therefore “to guarantee the preservation of its inherent values, all of which are at the service of improvement of individuals and society in general; to protect the integrity of sport competition” (7), in doing so “contributing to its credibility and legitimacy” (5). In this context, the autonomy of sport is “commonly justified as an important tool through which the values inherent to sport can be safeguarded from political, legal and, in the modern era, commercial influences” (8).

To safeguard their traditional systems of hierarchical self-governance, SGBs continuously engage in intensive lobbying towards political institutions such as the United Nations (9) or the European Union (EU) (10). In 2013, IOC President Thomas Bach told the United Nations General Assembly that the application of the “universal values and goals” shared by the IOC and the UN through a “universal law” depended on “[p]olitics respect[ing] … sporting autonomy” (11).

While the autonomy in international sport governance is “most evident and most vigorously defended within the Olympic movement” (12), it is also important to note neither the IOC Code of Ethics nor the Olympic Charter has produced “an extremely articulate definition of it” (13). In 2015, UNESCO excluded the term “autonomy of sport” from the revised 1978 International Charter of Physical Education and Sport, since it is “not yet sufficiently defined and would require further contextualization” (14). Beyond and within the field, the concept of autonomy was described as hard-to-define (15), far-stretching (16), myth (17), “deceiving veil for a brutal biopolitical takeover” (18), with its application that can be “dangerous to itself” (19).

The sport ecosystem is not carved in stone and is not immune to the reality of an ever-changing world (20). The claim to an autonomous organisational culture, with non-interference of external forces and international law, has been called the most far-reaching, and probably most disputed, principle of the Olympic Movement (1). It has been increasingly called into question over the past two decades in light of challenges arising from the professionalisation, commercialisation and globalisation of sport (21). While SGBs such as national or international federations are still associations by name and legal status, some of them have developed into multinational corporate monopolies in the global entertainment industry (22). These developments, together with the increasing number of stakeholders (commercial and political) involved, raise the question of whether the autonomy of sport in terms of self-governance is still appropriate and legitimate or whether more regulatory oversight is warranted “to ensure that sport as a business is still run for the love of the game and not just for the love of the money” (23).

Yet despite the importance of the concept, there is still no general consensus in academic discourse as to what the term actually means and how widely the principle is (supposed to be) applied in a changing world of sport. From the published academic literature, there has been no systematic attempt to structure the growing number of contributions. In acknowledging the lack of a structured research synthesis in sport policy/management domain, Dowling et al. (24) draw upon Forscher's (25) analogy of “*Chaos in Brickyard”* and outline the danger that “builders and bricklayers (researchers) might continue to produce studies (bricks) that would be thrown onto a pile of research without any consideration of how they contribute to a body of knowledge (edifices)” (p. 765). To counteract such a development within the scholarly discourse on sport autonomy and to reduce the research gap, the overall purpose of this paper is to systematically review the existing literature and knowledge on the concept and to thus provide “evidence” in order to understand how both researchers and practitioners have used the autonomy of sport concept (26).

The specific research objectives of this study are to: (1) systematically map out the extent and range of research on the concept of sport autonomy, (2) identify how the concept of sport autonomy has been defined within published and grey literature, (3) explore the autonomy in practice within international sport governance, (4) identify the regulatory frameworks and legal bases that govern the autonomy of sport, and (5) examine reported limitations of the concept of sport autonomy.

By offering insights into the context, action, content, and outcome of the autonomy of sport concept, as well as the challenges and opportunities arising from these processes, we make valuable contributions to the field of sport governance, politics, policy, and law. Firstly, the review advances the field of sport governance [i.e., governance understood as how organisations are led, controlled and regulated (27)] by organising and synthesising a currently fragmented body of research. In contrast to traditional literature reviews, the systematic approach of the scoping review allows us to conceptualise the contributions from various disciplines and identify gaps in the current knowledge. Secondly, in conducting this review, we make the first attempt at mapping the literature on the autonomy of sport holistically. Thirdly, based on Geeraert et al. (21) suggestion that autonomy should be understood as a multidimensional concept, we draw on existing dimensions of the autonomy of sport and contribute to theory by further developing existing conceptualisations. The overarching result of synthesising and mapping the literature is the development of a more comprehensive and nuanced understanding of the concept of autonomy within the governance of sport.

## 2 Methods

A scoping review was chosen for this study as “a rigorous and transparent method” to synthesise the body of knowledge on the concept of autonomy and discuss its characteristics in the field of sport governance by including a range of study designs in both published and grey literature (28, 29). Scoping reviews are an optimal tool to determine the scope and volume of a body of literature, as well as to identify available evidence and gain an overview of its focus when there is a lack of conceptual and analytical clarity (30, 31). Developing a scoping review serves as an audit process to create a structured approach to mapping the broad field of institutional studies in a given area (24, 32). Scoping reviews are increasingly becoming a go-to method for sport policy and management researchers across a broad range of topics in the sport management domain (33, 34, 35, 36).

As a specific methodological framework, this scoping review was guided by Arksey and O'Malley's (28) staged framework, which was elaborated by Levac et al. (29) and supported by the Joanna Briggs Institute (JBI) guidance for the development of systematic scoping reviews (37, 31). To increase methodological transparency, the Preferred Reporting Items for Systematic Reviews and Meta-Analyses extension for Scoping Reviews is used as a reporting guideline [PRISMA-ScR (26),], which provided a 20-point checklist (see Supplementary Material 1). Data from a systematic database search, supplemented by a systematic manual search of grey literature, is used to synthesise the body of knowledge on the concept of autonomy in sport. The following sub-sections describe the steps of this study based on five stages: (1) identification of the research question(s), (2) determination of relevant studies, (3) selection of studies, (4) data extraction, (5) collating, summarising, and reporting the results.

### 2.1 Identification of the research question(s)

In line with our research objectives, the main research question was formulated to guide the review: What role does the autonomy of sport concept play in the governance of sport? Based on this, the following sub-questions are addressed in the study: (1) What is the scope of literature on the autonomy of sport concept? (2) How is the concept defined in the records? (3) How is the concept applied in practice within the governance of sport? (4) What are the regulatory bases that govern autonomy of sport? (5) What are limitations of the concept? In line with the overarching objectives of a scoping review, this study will also identify potential future directions for research.

### 2.2 Determination of relevant studies

This study applied a two-phase search process consisting of a systematic database search supplemented by a systematic manual grey literature search.

#### 2.2.1 Phase 1

To identify relevant studies for a scoping review, we searched three major electronic databases: Scopus (Elsevier), SPORTDiscus (EBSCOhost), and Web of Science (Web of Science Core Collection). In addition, Google Scholar was included. These databases and a search engine were selected because they are comprehensive and multidisciplinary. According to the Cambridge English Dictionary, the terms “freedom”, “independence”, “self-governance”, “self-regulation”, “self-review”, “steering” are synonyms for “autonomy”. These concepts were included to capture as many autonomy concepts present in sport and to prevent the exclusion of potentially relevant records, which may have omitted the core term in their title, abstract or key words.

Following a couple of search strategy tests as a search validation procedure and the preregistering of the protocol at Open Science [i.e., a time-stamped, read-only version of the research plan submitted to a public registry prior conducting the search (38)], the lead author conducted the initial search on 22 May 2024, using the following search strategy with Boolean operators:

(TI^1^ autonomy OR self-regulation OR self-review OR self-governance OR freedom OR independence OR steering) OR (AB autonomy OR self-regulation OR self-review OR self-governance OR freedom OR independence OR steering) OR (AW autonomy OR self-regulation OR self-review OR self-governance OR freedom OR independence OR steering) OR (SU autonomy OR self-regulation OR self-review OR self-governance OR freedom OR independence OR steering) AND (TI sport^2^ OR “sport organi” OR “sport governing bod” OR “sport federation” OR “sport association”) OR (AB sport OR “sport organi” OR “sport governing bod” OR “sport federation” OR “sport association”) OR (AW sport OR “sport organi” OR “sport governing bod” OR “sport federation” OR “sport association”) OR (SU sport OR “sport organi” OR “sport governing bod” OR “sport federation” OR “sport association”).

The search strings differed for each database since the available fields and operators vary by database (see Supplementary Material 2A). Importantly, the key search terms remained the same for each database. The database searches were not restricted by date, subject or type.

The lead author was responsible for conducting a systematic database search: in total, the three databases and one search engine yielded a total of 9.147 records: Google Scholar: *n* = 300 (an *a priori* decision was made to review the first thirty pages of the search as it was considered unlikely that further screening would yield many more relevant records); Scopus (Elsevier): *n* = 4.978; SPORTDiscus (EBSCOhost): *n* = 3.224, Web of Science (Web of Science Core Collection): *n* = 645.

#### 2.2.2 Phase 2

As Mazzucco aptly noted (40), “issues related to the normative autonomy of international sport bodies were usually of purely academic interest”, yet these issues acquire practical significance. To give enough breadth to the consulted literature and “bridge the research-practice gap” (41), our search included not only academic but also grey literature [i.e., any documents not formally published through traditional academic channels like journals and books, such as statutes, internal regulations, white papers, policy documents, court rulings, reports and press-releases (42).] We grouped the diverse and heterogeneous body of material available outside the traditional academic peer-review process into (1) intergovernmental organisations (including regional), (2) courts, (3) selected stakeholders of the Olympic Movement and international football,^3^ (4) grey literature databases, and (5) targeted projects. A grey literature search plan incorporated a key search terms strategy, using the website's search box and a hand-searching method (see Supplementary Material 2B). The lead author, with the support of the research team, conducted a systematic manual search focused on grey literature through a series of targeted sources from relevant organisations and initiatives. Table 1 summarises the reviewed grey literature.

**Table 1:** Grey literature.

Category	Sources
Intergovernmental organisations (including regional)	Council of Europe, European Commission, European Parliament, UNESCO, UNODC
Courts	Court of Arbitration for Sport, European Court of Justice^a^
Selected stakeholders of the Olympic Movement and international football	ARISF, ASOIF, AIOWF, FIFA, IOC, UEFA
Grey literature databases	Olympic World Library, Open Grey
Targeted projects	IPACS, Play the Game SGO, SIGGS

ASOIF, association of summer olympic international federations; ARISF, association of IOC recognised international sports federations; AIOWF, association of international olympic winter sports federations; FIFA, fédération internationale de football association; IOC, international olympic committee; IPACS, international partnership against corruption in sport; SGO, play the game/sports governance observer; SIGGS, support for the implementation of good governance in sport; UEFA, union of european football associations; UNODC, united nations office on drugs and crime; UNESCO, united nations educational, scientific and cultural organisation.

^a^ We recognise that the interventions of European courts (or other bodies) revolve around the concept of autonomy that emanates from the European model of sport. Thus, by focusing on these rulings or decisions we tend to limit ourselves to discussing the concept of autonomy predominantly from European/Western perspectives.

### 2.3 Selection of studies

#### 2.3.1 Identification of studies *via* electronic databases

To exclude studies that are not relevant to our research questions, records were screened for relevance, first based on their title and abstract and then full text. The initial search yielded 9.147 total records, which were downloaded into Rayyan (i.e., an AI powered platform for systematic literature reviews). 1.717 duplicates were then removed from the list. For the first phase of screening, the lead author and the second author both independently screened the title and abstract of 7.430 remaining records for relevance based on the screener instructions and inclusion and exclusion criteria (see Supplementary Material 3). We further refined our search by focusing on English-language records only. We also deliberately chose not to delimit our timeframe to ensure complete coverage of the literature. Table 2 summarises the inclusion and exclusion criteria. To ensure the reliability of the selection process, Cohen's Kappa coefficient for inter-rater reliability was calculated after screening the first 100 records in Rayyan, resulting in k = 0,89, where a Kappa of greater than 0.8 is considered to represent a high level of agreement between the researchers (43).

**Table 2:** Inclusion and exclusion criteria for the scoping review.

Inclusion criteria	Exclusion criteria
Study-specific limits	
Sport-specific texts	Non-sport texts • Articles that do not discuss autonomy (and its variants) • Articles that do not discuss sport organisations (and its variants)
Date limits	
Up to and including the selected date of search (no lower date limit)	
Language limits	
In English	Not in English
Geographic limits	
All geographic areas	
Population limits	
• all sport organisations • intergovernmental organisations (including regional)	
Search items limits	
Includes search items in at least one of the following: research questions, keywords, title, or body of text	
Publication type limits	
• Journal articles and review articles, academic books, academic book chapters, conference abstracts, theses, empirical and conceptional studies • Grey literature (statutes and internal regulations, policy documents, press-releases, reports, white papers)	

Based on the screening of the titles and abstracts, 7.233 records were excluded as several search items also corresponded to other autonomy applications (e.g., autonomy-supportive teaching, or autonomy of the patient with the disability). If the relevance of a record could not be determined with certainty, it was subjected to a full-text screening. This left 197 records which were subject to independent full-text screening by the lead and second author. The study selection in a scoping review involves *post hoc* inclusion and exclusion criteria based on establishing the familiarity with the subject matter through reading the records (28). After the first phase, it was determined within the research team that for a “more expansive inclusion criteria” during full-text screening, the inclusion and exclusion criteria should be refined (30).

**Figure 1:**
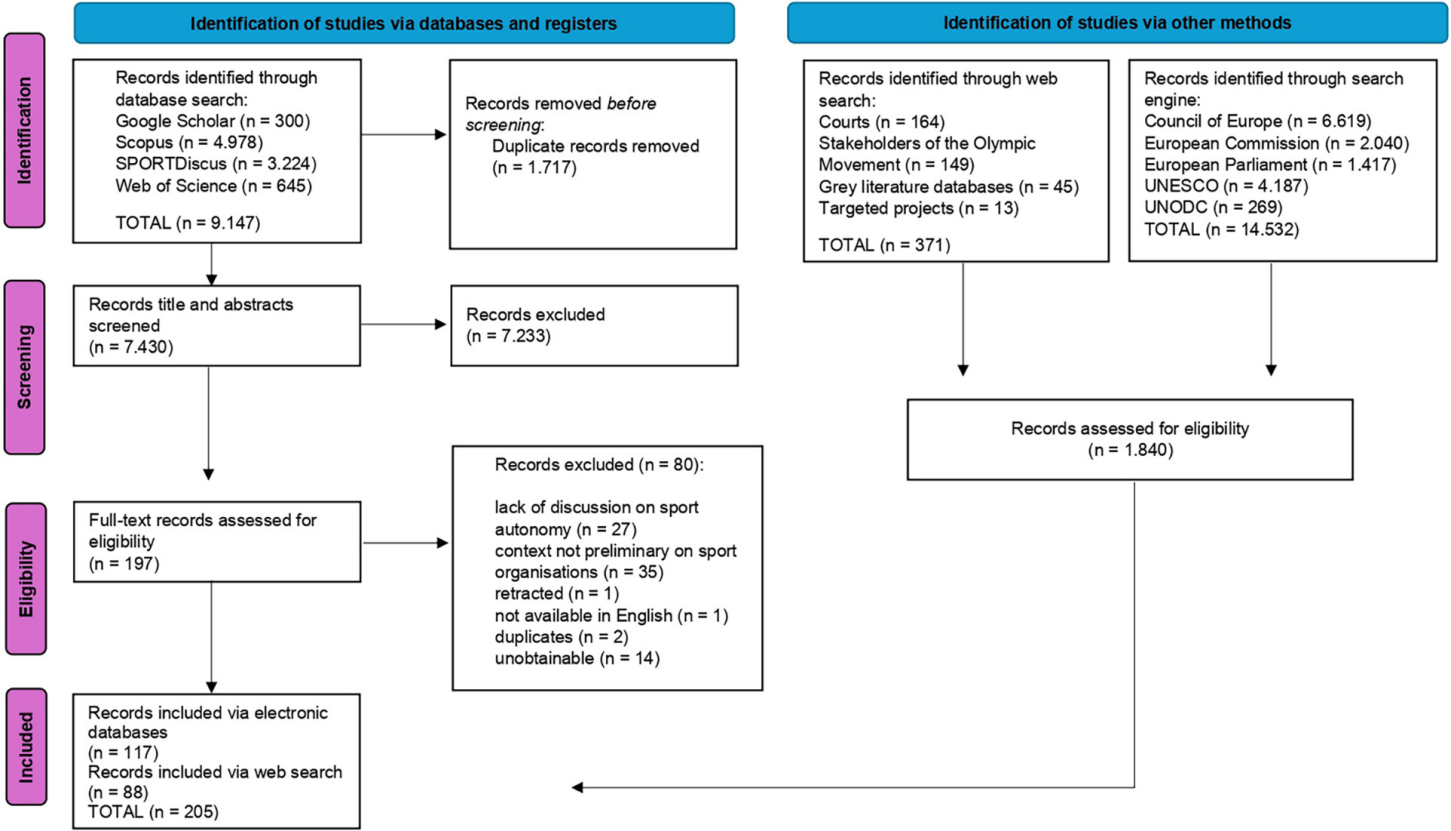
PRISMA flow char of the study selection process.

Of the 197 records screened, 80 were excluded due to the following reasons: record had no discussion of autonomy in the actual text beyond the title, abstract and keywords (*n* = 27), the context of the record was not primarily about sport organisations (*n* = 35), unobtainable (*n* = 14), duplicates (*n* = 2), retracted (*n* = 1), not available in English language (*n* = 1). In the end, this resulted in 117 records being accepted for a further phase: data extraction (see Supplementary Material 4A). Figure 1 illustrates the PRISMA flow chart with overview of the screening process and reasons for full-text exclusions.

#### 2.3.2 Identification of studies *via* web search

A systematic manual grey literature search yielded 14.532 records. For the manual search of grey literature databases, the key search items were comprehensive to capture the most relevant literature in the first thirty pages of search hits (149). Despite limitations in the search engine functionality of some of the organisations' websites, records were searched in the relevant sections of the website. A purposive sampling approach was employed to identify relevant rulings of the Court of Justice of the European Union (CJEU). Rather than relying solely on organic citation chains, the search deliberately centred on known landmark cases, especially the most recent ones – International Skating Union (ISU), European Superleague Company, Royal Antwerp Football Club, and Diarra. These high-profile decisions served as a guiding framework for selecting additional cases referenced within the articles, thereby ensuring targeted coverage of the most influential sport-law rulings. The full-text screening of the records was conducted individually by the lead and second authors, resulting in 88 records accepted for the subsequent phase (see Supplementary Material 4B). This means that a total of 205 records were further processed (see Figure 1).

### 2.4 Data extraction

The fourth stage of the scoping review involved data extraction from the identified records from the search process to inform the research objectives and questions. According to Arksey and O’Malley (28), data extraction is a technique for organising and interpreting data into qualitative themes. Following the principles of data extraction, authors agreed on a standardised data extraction form and guidance for the form (44) (see Supplementary Material 5). Data extraction was carried out using Microsoft Excel (Redmond, Washington, USA) and involved collecting the following study characteristics on all records: author names, article title, year of publication, country of the university affiliation of the first author, publication type (e.g., journal article, book chapter, etc.), country of study context, type of sport examined, research design (empirical, non-empirical), theoretical framework, data collection strategy (e.g., survey, interviews, etc.), dimension autonomy of sport as defined by Geeraert et al. (21), (political, legal, financial, pyramidal). In addition, we also extracted: definitions of autonomy, limits and prerequisites to autonomy, developments affecting autonomy, and future research reported. The extraction was conducted by the lead author. The research team met regularly throughout this stage to ensure accuracy and consistency of the data extraction process.

### 2.5 Collating, summarising, and reporting the results

The fifth stage reports the findings through descriptive frequency analyses and qualitative content analysis (28). The frequency analysis provides a descriptive numerical summary of the nature, extent, and distributions of the studies reviewed for a scoping study (29). Our frequency analysis includes the year of publication, type of publication, country of first author and country of study context, research design, theoretical framework, sport type, type of organisations, definitions, and dimensions of autonomy. Following other scoping reviews [e.g., (45)], we focused the frequency analysis exclusively on academic records. These allow for clearer analysis of key study characteristics, as the variables are more consistently reported in scholarly literature and are essential for conducting comprehensive frequency analyses. Grey literature, while valuable for contextual insights in a qualitative content analysis, often lacks the uniformity required for robust quantitative synthesis. Of the 88 records identified by the web search, eight were found to be academic in nature, bringing the total to 125 records included in the frequency analysis (see Supplementary Material 6).

Consistent with the suggestion of Peters et al. (31), a qualitative content analysis was then applied to synthesise the texts. An inductive orientation to the data was employed opening the analysis up to the identification of patterns emerging from the data. Following the example of the JBI Scoping Review Methodology Group (44), the research team adopted the following process of conducting the qualitative content analyses: (1) immersion in data, (2) inductive extraction and analysis, (3) open coding, (4) develop coding framework, (5) extraction and organising, and (6) categorisation. Specifically, the first author reviewed the information contained in the chart form for all 205 records, extracted data according to the specific research objectives.

## 3 Results

### 3.1 Frequency analysis

To address research question (1), a summary of the selected records is presented with the corresponding outcomes of the frequency analyses to provide a big picture view of the autonomy of sport literature.

#### 3.1.1 Year of publication

**Figure 2:**
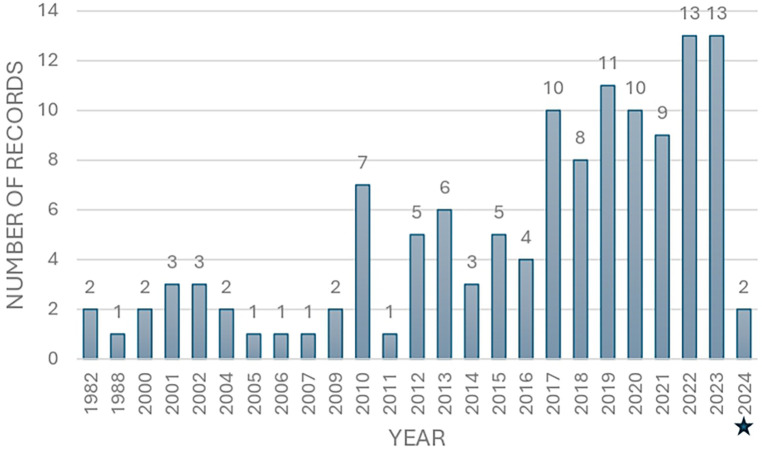
Frequency of records examining the autonomy of sport. The figure includes studies from electronic databases and grey literature search engines. *For 2024, only records available before May 21 were included.

The year of publication analysis highlighted the recent growth of the research on the autonomy of sport. Figure 2 illustrates that the records were published between 1982 and 2024, with almost 70% (*n* = 85) of the selected records were published since the second half of the last decade (2015–2024). The most frequent years are 2022 and 2023, each with 13 publications (10.4% of the total). This means that the records used in our analysis can be considered as the recent contributions to the field of autonomy of sport. While there were fluctuations in the numbers of records on an annual basis, a noticeable upsurge was observed in 2017 (*n* = 10).

#### 3.1.2 Academic field

The 125 records included 87 journal articles, 19 book chapters, ten books, eights reports, and one (master) thesis. The 125 records were published in 49 different journals, reflecting the multifaceted nature of the autonomy of sport not only in disciplinary depth but also in multi- and interdisciplinary width. These journals cover a wide range of academic fields, including law, policy, and governance, with some having significantly opposing scopes. Approximately 24% were published in three leading peer reviewed journals, including *International Sports Law Journal* (*n* = 16), *International Journal of Sport Policy and Politics* (*n* = 7), and *International Sports Law Review Pandektis* (*n* = 7). There are two additional publishers of note that contain approximately 11% of all records: Routledge (*n* = 7), Springer Nature (*n* = 7). The reports were published by a variety of sources, including Play the Game/Danish Sport Institute (*n* = 5), Council of Europe (*n* = 2), and European Parliamentary Research Service (*n* = 1).

#### 3.1.3 Country of first author's university affiliation

**Figure 3:**
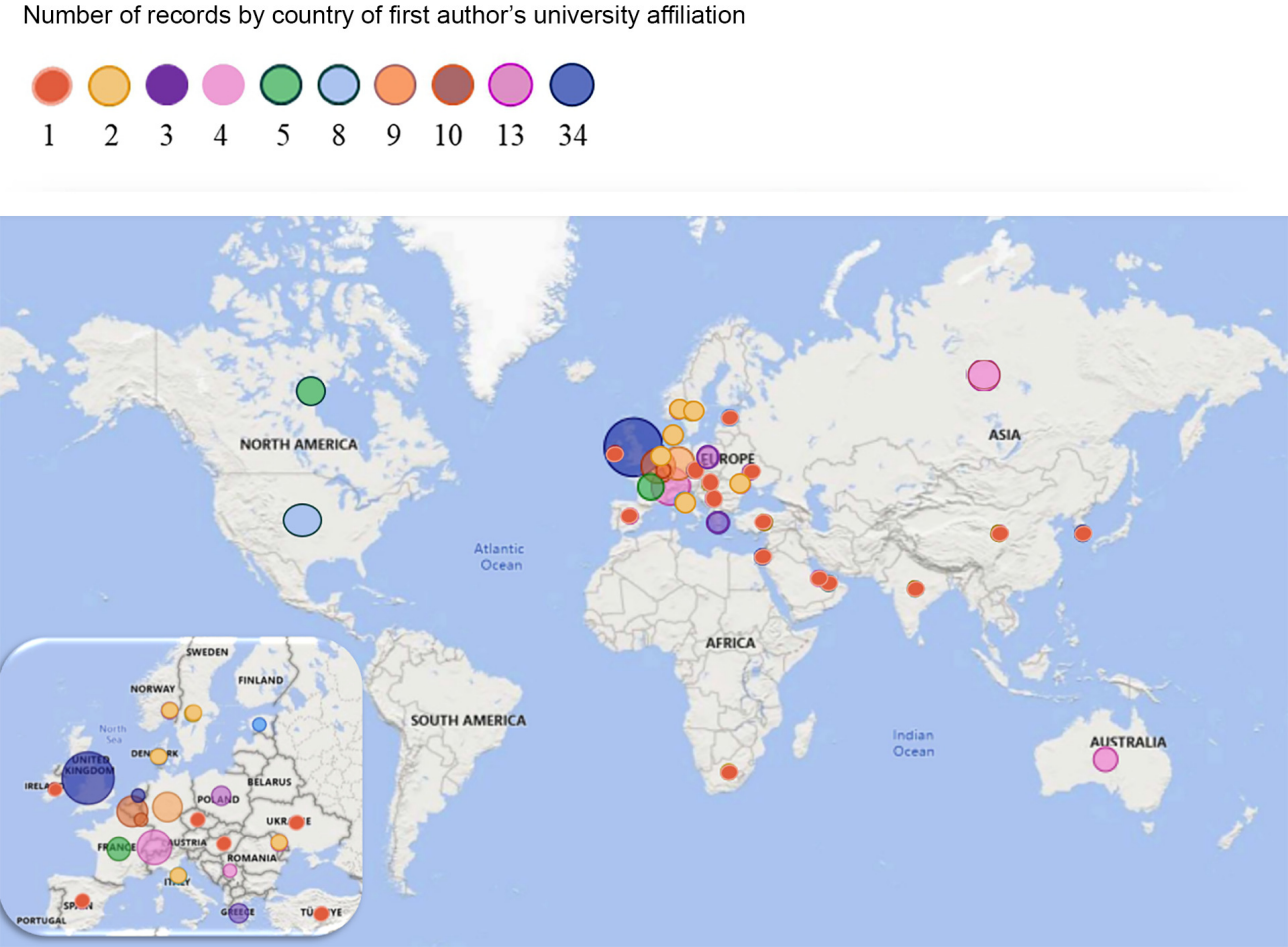
World heat map showing the number of included records conducted in each country.

Records originated in 33 countries, but most studies were carried out in the United Kingdom (*n* = 34; 27.2%). Other countries with multiple studies were Switzerland (*n* = 13; 10.4%), Belgium (*n* = 10; 8%) and Germany (*n* = 9; 7.2%). The findings point to the Eurocentricity of the research and the dominance of western European ways of seeing, describing and embracing the autonomy of sport, which are not necessarily representative of the whole constituency. Figure 3 illustrates world heat map showing the number of included records conducted in each country. The most prolific researchers were identified as Borja García (7), Ken Foster (6), Jean-Loup Chappelet (5), Arnout Geeraert (5), and Stephen Weatherill (3) with his book-length treatments of autonomy of sport.

#### 3.1.4 Country of study context

The context of the study refers to the country where the autonomy of sport was examined. Research was contextualised in more than 21 different countries, with most records articles having multicountry contexts (*n* = 77). As such, García and Meier (46) explore “autonomy” in the relations of the IOC, National Olympic Committees (NOCs), and national governments in Botswana, Guatemala and Sri Lanka. Fischer et al. (47) contrast the interventionist model of France with the more liberal model of Germany, discussing autonomy in terms of regulatory powers and state influence. Zintz and Gérard (7) present the overall mean and national scores on autonomy and accountability of Belgium, Germany, Lithuania, Luxembourg, Portugal, Slovenia, and Turkey [results collected through the EU project “Support the Implementation of Good Governance in Sport (SIGGS)”]. A considerable number of records was contextualised in the European Union (*n* = 22). One or two studies were conducted in the remaining 21 countries. Table 3 summarises the frequency of studies by country of study context.

**Table 3:** Number of records by country of study context.

Country of study context	Frequency	Percent
Multi-country	77	61.6%
EU	23	18.4%
Moldova	2	1.6%
Russia	2	1.6%
Switzerland	2	1.6%
Belgium	1	0.8%
Botswana	1	0.8%
Canada	1	0.8%
Denmark	1	0.8%
France	1	0.8%
Germany	1	0.8%
Greece	1	0.8%
Hungary	1	0.8%
Israel	1	0.8%
Italy	1	0.8%
Poland	1	0.8%
Qatar	1	0.8%
Scotland	1	0.8%
Serbia	1	0.8%
South Africa	1	0.8%
South Korea	1	0.8%
United Arab Emirates	1	0.8%
USA	1	0.8%
Yugoslavia	1	0.8%
Total	125	100.0%

#### 3.1.5 Research design

The studies employed a range of research designs. Most studies (*n* = 104, 83.2%) were non-empirical [i.e., theoretical or conceptual] in nature. An empirical approach was used in 21 (16.8%) records with the most common data collection strategies being semi-structured interviews, and either sociological questionnaires or descriptive surveys. Table 4 summarises the data collection methods in empirical studies.

**Table 4:** Data collection methods in empirical studies.

Data collection method	Records
Interviews & document analysis	Choi (48), García et al. (15), García and Meier (46), García and Weatherill (49), Geeraert (50, 51), Minikin (52), Winand et al. (53)
Public documents	Abrutyn (54), Harris et al. (55), Szatkowski (56)
Surveys	Wickstrøm and Alvad (57), Yaghi and Almutawwa (58), Zintz and Gérard (7)
Interviews & surveys	Ioannidis (59), Zeimers et al. (60)
Survey & document analysis	Geeraert et al. (16)
Systematic review	Thompson et al. (61)
Documents, interviews & observations	Budevici-Puiu and Manolachi (62)
Questionnaire, interviews & documents	Geeraert (63)
Questionnaire & observation	Budevici-Puiu et al. (64)

#### 3.1.6 Theoretical frameworks

In terms of theoretical frameworks, the analysis revealed that 93 (74.4%) records did not explicitly state the use of any theory. In total, 32 (25.6%) different theoretical frameworks were identified, with some records using two or more theories. To name but a few: the principal-agent theory was used to describe the hierarchical relationships between national governments and sport organisations, where governments act as principals and sport organisations as agents, that enables the agent to serve its own interests at the expense of the principal which could result in imperfect agent behaviour because the agent can exploit its autonomy and minimise its efforts on behalf of the principal (1, 65); compliance theory was utilised to analyse the self-regulation initiatives of sport organisations (51); institutional theory was employed to explore how external pressures from stakeholders and regulators influence sport organisations to adopt specific practices and adapt to changes in order to meet perceived performance determinants and legitimacy expectations (53, 58).

#### 3.1.7 Sport type

While 108 (86.4%) records did not focus on a specific sport and either used a multisport approach, the remaining records investigated autonomy of sport focusing on football (*n* = 16, 12.8%) and baseball (*n* = 1, 0.8%). Dolbysheva (66) examined the autonomy of non-Olympic sport. The emphasis on football is consistent with football's cultural and economic significance, particularly within Europe, where the Union of European Football Associations (UEFA)'s activities have been focal point for legal scholars since the landmark Bosman ruling in 1995.

#### 3.1.8 Type of organisation

The records examined different types of organiations that make up the Olympic Movement, such as international multi-sport governing bodies and/or international sport organisations (*n* = 61), national multi-sport governing bodies and/or national sport organisations (*n* = 22). Approximately one third of the records (*n* = 42) combined national and international sport organisations. These findings highlight that the autonomy of sport is shaped by the interplay between international organisations. The IOC imposes structures and policies through mechanisms, such as the recognition of National Olympic Committees, which needs to be part of the national sport system, yet it must remain politically autonomous from the government and the state.

#### 3.1.9 Definitions and dimensions of autonomy

Almost half of the records (45.6%, *n* = 57) did not define autonomy, what underlines the need to provide more conceptual clarity. 68 records (54.4%) provided at least one definition of autonomy or a closely related term (i.e., freedom, independence, self-governance, self-regulation, self-review, steering). We collected data about how the number of records featured the dimensions of the autonomy of sport [political, legal, financial, pyramidal – as defined by (21)] in the full text. Despite the analytical distinction, the findings highlight that the borders between autonomies in the practice of sport governance are rather ambiguous. The politico-legal records (32.8%, *n* = 41) place sport governance in the social realm around politics and a normative context, focusing on various normative sources exterior to sport that have an impact on the autonomy of sport. Table 5 summarises this analysis.

**Table 5:** Frequency of dimensions of autonomy—political (Pol), legal (L), financial (F), pyramidal (Pyr).

Dimensions	Freq.	Percent
Pol, L	41	32.8%
Pol, L, F, Pyr	35	28.0%
L	22	17.6%
Pol	10	8.0%
Pol, L, F	5	4.0%
Pol, L, Pyr	4	3.2%
F	2	1.6%
Pol, F	2	1.6%
Pol, Pyr	2	1.6%
Pol, L, F	1	0.8%
Pol, L, P	1	0.8%
*Total*	125	100.0%

### 3.2 Qualitative content analysis

Based on the established research objectives of our review, the analysis identified patterns in the extracted data and generated key topic areas (see Table 6). We now discuss our findings related to research questions (2) to (5) in turn. Where applicable, future directions for research are outlined.

**Table 6:** Topic areas of sport autonomy records.

Research objective	Example authors	Example topic areas
Definition of the concept of sport autonomy	Allison and Tomlinson (17), Chappelet (5, 3), Dolbysheva (66), García and Meier (46), Girginov (67), Panagiotopoulos and Kallimani (68), van der Walt (69)	• Evolving nature of autonomy of sport • History of autonomy of sport • Normalisation committees (in case of non-compliance) • Role of culture in shaping the meaning and practices of autonomy • Western origin of autonomy
Application of autonomy in practice	Abanazir (70), Geeraert et al. (21), Scheerder et al. (65), Winand et al. (53)	• Cross-national perspective • Degrees of autonomy • Dimensions of autonomy • Shift from hierarchical to networked governance • Ties between NOCs and national governments
Regulatory frameworks and legal bases	Baddeley (71), Bruyninckx (72), Coleman (73), Duval (74), Foster (23), González (75), Kornbeck (76), Lenskyj (77), Lewandowski (78), Vieweg (79), Weatherill (80)	• Compliance with human rights standards • Compliance with national laws • Court of Arbitration for Sport • EU law • External oversight to audit sports organisations • Implications of Court of Justice of the European Union rulings • International pubic law • Juridification of sport • Legal challenges from national courts • *Lex sportiva* • Specificity of sport • Swiss regulatory framework
Limitations of the concept of sport autonomy	Girginov (81), Colucci and Geeraert (82), Chappelet and Mrkonjic (83), Jedlicka (12), Næss (84), Zeimers et al. (60), Zintz and Gérard (7)	• Governmentalisation and politisation of sport • Role and intervention of the intergovernmental organisations (e.g., UN/EU) • Financial (over) dependency on government funding • Financial dependency on television rights and corporate sponsorship • Corporate social responsibility • Threats to the integrity of sport • Social dialogue among stakeholders/stakeholder engagement • Quantification of good governance

#### 3.2.1 How is the concept defined by record authors?

Various definitions have been proposed and deliberated upon by academics and practitioners as relevant to the reflection on the autonomy in the international sport governance. Table 7 provides a selected overview of the variety of definitions, illustrating the breadth of the applications of the autonomy of sport. Record authors have approached the concept of autonomy in varied ways, often adopting definitions based on their respective research agendas or the phenomenon being studied.

**Table 7:** Examples of the autonomy of sport definitions.

Author	Definition
Budevici-Puiu and Manolachi (62)	“the autonomy of sport is an essential feature of the sports sphere, reflecting the decentralization of management. In this field, the normative, institutional-structural and organizational independence of the financial, economic, political and ideological activity of the sports sphere from public authorities, political organizations, religious associations and business organizations, independence from authorization, interference and pressure from them” (p. 463).
Chappelet (5)	“The autonomy of sport is, within the framework of national and international law, the possibility for non-governmental, non-profit-making sports organizations to: establish, amend, and interpret rules appropriate to their sport freely, without undue political or economic influence; choose their leaders democratically, without interference by states or third parties; obtain adequate funds from public or other sources, without disproportionate obligations; use these funds to achieve objectives and carry on activities chosen without severe external constraints; draw up, in consultation with the public authorities, legitimate standards proportionate to the fulfilment of these objectives” (p. 49).
Harris et al. (55)	“autonomy is a form of cultural capital, created by sport in the early 1900s and vehemently sustained to this day, to enable sport to manage its own affairs and to protect it from external interference. The principle of autonomy means that the field is entirely reliant upon each agent in the field being accountable and fulfilling their regulatory responsibilities” (p. 365).
Rook et al. (85)	sport's autonomy is “an enabler of equal treatment and non-discrimination and the universal application of the Olympic values and principles, regardless of political regime, legislation, culture and religion” (p. 94).
van der Walt (69)	“autonomy refers to a sporting institution's claim to an exclusive and final right of determination and decision and an exclusive responsibility in regard to its own, unique domestic affairs” (p. 30).

One of the most comprehensive explanations of the autonomy of sport comes from Geeraert et al.'s (21) re-examination of Chappelet's (5) earlier work. Chappelet (5) initially proposed that sport autonomy is a multidimensional concept encompassing pyramidal, psychological, political, legal, and financial aspects (pp. 29–33). In their revised framework, however, Geeraert and colleagues excluded the psychological dimension and redefined the autonomy of sport through four key dimensions: “political autonomy”, “legal autonomy”, “financial autonomy”, and “pyramidal autonomy”. Political autonomy is defined as “the historic and path-dependent autonomy of a [SGB] to fulfil its primary function built upon freedom of association, without being subject to political interference from public authorities”. For its part, legal autonomy is “the private autonomy of a [SGB] to fulfil its primary function with a legal impact at national or at international level, determined and confined by the legal framework imposed by public authorities”. Financial autonomy concerns “the capacity of a [SGB] to fulfil its primary function, while not relying on external public investment, internal systemic resources or sponsoring from a single commercial partner”. Finally, pyramidal autonomy is “the autonomy of a [SGB] to fulfil its primary function within a hierarchical pyramidal system” (21).

Recognising the clarity and solidity of Geeraert et al.'s dimensions of autonomy (21), Abanazir (70) argues that moral autonomy flows into other dimensions of autonomy “so that the sport association keeps the state and the market at bay when the normative order's aims and interests are under threat” (p. 18). Geeraert (86) also addresses the internal and external elements of autonomy, where “internal autonomy” presents a scenario where stakeholders have more power to shape the policies of the organisations to which they belong, while “external autonomy” suggests that the state and the market have the potential to influence their actions (p. 255).

To explain the evolving nature of the autonomy principle within the Olympic Movement, some of the record authors discuss the Western origins of the autonomy of sport and consider how the concept of the autonomy of sport is masquerading as universal, travelling around the world on the back of the IOC, which is culturally and geographically European, while shaping the way non-Europeans experience sport (81). Scheerder (1) utilised a socio-politological approach to analyse the history of autonomy in modern sport that is rooted in the development of organised sport in the eighteenth to nineteenth century England. This historical foundation aligns with Allison and Tomlinson's (17) note that “the long established traditions in British sport of pluralism and voluntarism created a barrier to state intervention in sport” (p. 117). Expanding on this, Geraert et al. (21) and Chappelet (3) emphasise that sport autonomy is a fundamentally Western concept, rooted in the idea that sport is part of the recreational activities of free citizens who can form their own associations and set their own rules. Historically, this principle emerged when sport was considered politically inconsequential and supported by a civil society with sufficient social capital for voluntary organisation (13).

Over the second half of the 20th century, the Olympic Movement has grown into a dynamic industry that attracts significant political interest, reaching countries that lack developed civil societies and human freedoms. This global expansion raises important questions about how autonomy is understood and practiced in different cultural and political contexts. Scholarship stemming from Third World Approaches to International Law (TWAIL) highlights the role of culture in shaping the meaning and application of autonomy, arguing that Western interpretations do not necessarily align with non-Western governance structures and traditions (87). In this context, the IOC imposes structures and policies through mechanisms such as the recognition of NOCs. These NOCs are required to be integrated into the national sport system while maintaining autonomy from the state. Balancing compliance with the Olympic Charter and the national laws creates a complex dynamic of policy transfer and organisational tensions. Foster (88) emphasises that “these conflicting pressures can leave national sporting associations caught in the middle” (p. 49). Girginov (67) criticises the “homogenisation of diversity” in the governance practices and “cultural imperialism” where so-called Western values placed above others and at the expense of other dynamics.

The role of culture in shaping the meaning and practices of autonomy was highlighted in the studies that looked at how the IOC imposes the autonomy of sport as a “governance transplant” that originated in the Global North to the countries in the Global South without necessarily appreciating the difference in the way politics or political systems are structured in these countries (46, 13). This idea takes inspiration from the debate about “legal transplants” in comparative legal research, which refers to the application of laws and norms designed in a particular legal context and applied to a different environment for which the norms were not necessarily designed (89). In international sport governance, the Eurocentricity of such transplants played out in a variety of contexts leading to instances where the IOC has banned associations for not following imposed standards. Following the cases of non-compliance, there is an increasing number of normalisation committees' intervention where SGB temporarily takes control of a national sport governing body that fails to comply with the SGB's constitution (e.g., Statutes, or Charter, or Memorandum and Articles of Association) (90, 68, 69).

While the concept of autonomy may hold significance and value within democratic states, its implementation in authoritarian regimes, where freedom of expression is either non-existent or severely restricted, poses a considerable challenge (58). The autonomy of sport is difficult to justify outside the Western world. In 2017, Play the Game made a 'sports autonomy index' which showed that 14 per cent of NOCs were directly controlled by people with positions in government (57). A significant proportion of Asian NOCs were found to be under the direct oversight of individuals holding government positions, with one in three (36.4%) of these entities being subject to such direct control. Taken together, these perspectives reveal tensions between the traditional Western conceptualisation of autonomy and the evolving global realities of sport governance. It begs the question as to whether it is possible to have a universal definition of autonomy in the vastly diverse socio-cultural and political contexts that international sport governance finds itself in, and what that might lead to.

In light of the aforementioned, future research may explore approaches that can accommodate the diversity of structures and actors in international sport governance, thereby enriching the international academic dialogue and increasing the theoretical and empirical knowledge of comparative sport governance and policy far beyond the Western academic context and the Global North. Of particular importance is the evaluation of the relationship between NOCs and national governments in countries of the Global South, with a particular focus on the extent to which the concept of the autonomy of sport can be effectively implemented in these cases. Academic research on sport governance and policymaking should aim to develop governance models that are sensitive to local contexts and cultural diversity.

#### 3.2.2 How is the concept applied in practice within the governance of sport?

A dominant focus on a broad interpretation of the autonomy of sport does not do justice to the autonomy in practice of international sport governance, which is much more diverse than the constitutions of SGBs take into account. The best way to explore the application of autonomy is by distinguishing it from, at least at first sight, similar concepts. For instance, legal scholars use functional autonomy to reflect the special character of sport and the capacity to operate independently in their specific domain (80), disciplinary autonomy as SGB's enabler to initiate sport investigations (91), while regulatory autonomy refers to the power of SGBs to implement regulations (73, 78). Record authors made several distinctions in order to focus on internal and external of autonomy, such as internal associative (47) or organisational autonomy (92) and transnational autonomy of global sport (85) respectively. Ponkina (93) suggests the analytical distinction based on the nature of the autonomy of sport as normative, institutionally structural, organisationally active, ideological (politically ideological, religiously ideological), and financial.

Beyond these distinctions, application of autonomy varies across national contexts. A cross-national comparative studies draw on the structure of and the relationships between non-governmental sporting bodies and national governmental sport authorities. For example, Szatkowski (56) between distinguishes interventionist and non-interventionist models of sport regulation. Scheerder et al. (65) explain cross-national differences based on types of national sport systems: social, missionary, bureaucratic, and entrepreneurial configuration. Chappelet (5) distinguished four categories of NOCs depending on the degree of political independence: (1) politically independent NOCs with substantial financial resources; (2) politically independent NOCs without substantial financial resources; (3) NOCs controlled by government on both a financial and political level; (4) so-called “fantasy” NOCs, created for symbolic participation in the Olympics. This classification recognises the great heterogeneity among NOCs underscoring the impact of financial and political dynamics shape the operational realities.

**Figure 4:**
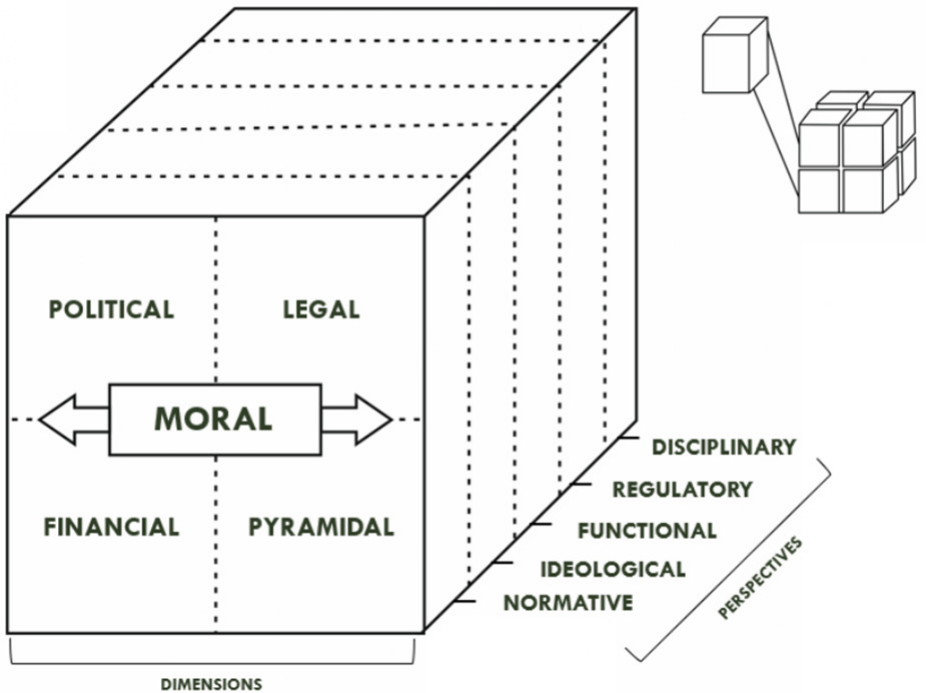
Sport autonomy cube.

Figure 4 presents our “Sport Autonomy Cube”, which represents a synthesis of the conceptualisations of sport autonomy, based on our findings. The cube illustrates that autonomy is multidimensional and interconnected and distinguishes between its dimensions and perspectives. The autonomy of sport requires a sophisticated deconstruction of its multiple dimensions and a conceptual understanding of its perspective in selected contexts.

Rather than viewing sport autonomy as a binary of “autonomous” and “non-autonomous”, Abanazir (70) offers an understanding of the concept of sport autonomy as a spectrum, ranging from full dependence on the state or market (−1) to full independence (1), with most organisations operating in a semi-autonomous state (0) (pp. 15–16). The emphasis on the term “semi-autonomous” provides a picture in which the focus on the application of autonomy in international sport governance practice is seen as a spectrum. This indicates that autonomy is a fluid concept rather than a static one. Autonomy is complex, therefore it needs to be studied in the dimensions of the interlinked autonomies, encompassing the varying degrees of autonomy.

The evolving nature of autonomy in practice of international sport governance provides a rich area for future research, with a focus on exploring the interplay between sport, politics and market forces. A deeper examination of the dynamics of stakeholder relationships is needed to understand their impact on the autonomy of sport. As well as exploring stakeholder democracy as a means of enhancing the legitimacy of SGBs.

#### 3.2.3 What are the regulatory bases that govern autonomy of sport?

An understanding of the sport sector is predicated on an understanding of who governs it. It is vital for anyone researching or working in the sport sector to understand where the autonomous power to govern and regulate a sport lies. This autonomy is shaped by a legal framework that regulates the private order of the Olympic Movement through applicable national and transnational laws. These regulatory bases, combined with political autonomy that emphasises the need to minimise state interference, create a context in which the primary functions of the SGBs relate to the exercise of legislative, executive and judicial authority over sport and its internal affairs (94).

##### 3.2.3.1 Legislative authority

SGBs have a clear legislative function as the guardian of the “laws of the game”, but these rules need to include “substantive criteria and detailed procedural rules” ensuring that they are: *transparent, objective, precise and non-discriminatory* [(95), Superleague, para. 147 (96); ISU, paras 133–136]. As such, the legislative authority of the SGB constitutes the power to amend its statutes and/or to adopt rules and regulations governing the conditions of play of the sport, and is vested in the members of the SGB, who meet together as a “Congress” or “General Assembly” (94). Garcia and Weatherill describe “specificity” as the “next best” argument of SGBs after autonomy, “autonomy is a claim to immunity. Specificity is a claim to have the law moulded in application to meet sport's special concerns” (p. 248). The autonomy and specificity of sport allowed the IOC to “perceive itself as a law unto itself” (97).

The IOC and SGBs are subject to limited binding law provisions at the international level and at the national level. The overarching relationships between public international law and sport governance are established through international conventions and resolutions that promote cross-sector cooperation by intergovernmental organisations in a way autonomy is not compromised. Examples of such conventions range from public order issues [hooliganism and spectator violence (98), corruption (99)] to integrity threats [doping (100), match-fixing (101)].

Based in Switzerland and operating under private law provisions of the Swiss Civil Code, the IOC and most SGBs are primarily governed by the legal framework of their country of domicile. Most of them remain subject to Swiss regulatory requirements and, by virtue of arbitration clauses enshrined in their statutes, rely upon the CAS and their internal judicial bodies as the principal forum for dispute resolution. Consequently, the authority and autonomy of SGBs are rooted in the Swiss legal system (102). Baddeley (71) discusses the extraordinary autonomy of sport bodies under Swiss law that derives from the liberal legislations in Switzerland governing associations and arbitration and from their equally liberal application by the courts. Kornbeck (76) elaborates on the approach of Swiss courts and the latitude left for SGB's self-regulation under Swiss law. Switzerland, home to numerous international sport bodies, is known to have more accommodating legislation in this area than many other countries. Di Marco (103, 104) defines Switzerland as a “legal paradise” for SGBs since Swiss law guarantees property and tax privileges for the optimal regulatory functions of the most SGBs. However, criminal immunity is over as Switzerland passed Federal Law on Amendments to the Criminal Code in 2015. The FIFA corruption scandals led the Swiss government to confining the legal autonomy of Swiss-based SGBs and amending the Unfair Competition Act and the Swiss Criminal Code, which were not applicable before.

##### 3.2.3.2 Executive authority

Between the general meetings of SGB's member in “Congress” or “General Assembly”, its powers are carried out by an “Executive Committee”, “Executive Board”, or “Council”, which consists of individuals who were elected by the member federations during a previous general meeting (94). In this regard, there is critical research on how European dominance has, to some extent, enabled the concentration of both power and resources in the hands of mostly European actors governing the Olympic Movement (16). In addition, the members of the Executive Board/Council are subject to extensive legal and ethical obligations in the exercise of their powers. Failure to comply with these obligations is often subject to legal challenge, most often on the grounds that they have followed an unfair procedure, failed to comply with their own statutes or applicable law, or exercised their discretion irrationally.

A set of records highlight the complexity of the application of conflict of interest in the boards of sport organisations. Bundling regulatory and political functions manipulates electoral democratic processes and breaks an assumption that elected representatives put the organisation's interests before their own and that they always act in the best interests of the members (52). Against such accumulation of roles, a set of records interested in adequate check on abuses of autonomy, internal checks and balances, and check on the rationality of SGBs' policies (73, 105). For example, Chappelet (106) proposes to draw up the “Lausanne Convention” to provide an international legal framework for a body to audit sport organisations. Weatherill (107) proposes an external oversight where good governance monitoring could be delegated to a European or international supervisor. In addition to this non-profit/public third-party assessment, the records consider institutional [e.g., ASOIF, independent integrity unit] and commercial [e.g., auditors] ways of monitoring (72). Scholarship observes a trend to place the executive authority of the SGB in the hands of an operationally independent integrity unit of the SGB, reporting to a separate board composed of individuals who are independent of the SGB. Despite the increased juridification of sport, it is more common to find that the main ambition of SGBs is to isolate their practices and activities from legal supervision, or at least to adapt regularity bases to legal rules to sporting needs (108). The records authors address how and to what extent an autonomy for sporting practices can be extracted (79, 80).

##### 3.2.3.3 Judicial authority

The international sport governance has established its own legal system, which empowers it to adjudicate disputes within its own network and in accordance with its own set of regulations [i.e., sporting rules] (109, 110). The system of sport dispute resolution has facilitated the development of a comprehensive body of global/transnational sport law, known as *lex sportiva* (74, 111, 112). In this context, *lex sportiva* acts as a “legal buffer” for the specific system of autonomous rules and regulations of SGBs (97).

Sport justice structures itself around a system that limits external interference, thereby facilitating *de facto* and *de jure* the autonomy of sport and allowing the international sport governance to be conceived in practice as a “legal system” (113). In this system, judicial authority is exercised both internally by the quasi-judicial bodies of SGBs and externally by the Court of Arbitration for Sport (CAS) (114). Most SGBs recognise the CAS as the only authoritative body to review their internal decisions. However, questions often arise concerning the transparency, independence and quality of the CAS review of SGBs decisions (74, 115). Transparency issues include the need for public hearings in disciplinary cases and greater clarity in CAS finances. Questions about CAS' independence challenge whether it serves as a truly accountable mechanism. Additionally, concerns about the substantive quality of CAS reviews highlight cases like Caster Semenya's, where CAS failed to properly assess the compatibility of World Athletics rules with human rights, highlighting insufficient proportionality analysis (116). In January 2025, in the opinion by Advocate General Ćapeta of the CJEU, Tamara Capeta challenges the authority of CAS. In the Seraing case, Capeta suggests that CAS awards should be challengeable in EU national courts under EU law, signalling a shift toward greater judicial oversight of sport governance (117).

Legal scholars tend to be more critical, in some cases even arguing that hiding behind the idea of autonomy was simply a method of avoiding accountability and keeping regulatory bodies such as the EU and the CJEU at bay (118, 119, 107). Numerous rulings of the CJEU – the most famous being the 1995 Bosman ruling – have highlighted the difficulties of reconciling sport's specific system of autonomous rules and regulations with national legislation and/or European directives (107). For example, in 2023, the CJEU has ruled that the UEFA contravened EU law when they used their regulatory autonomy to impose sanctions on clubs wishing to take part in a breakaway “European Super League”, set up in competition to UEFA's Champions League (120); in 2024, the CJEU arrived at the conclusion that some of the FIFA's players transfer rules are incompatible with EU law (102). The CJEU acknowledges the legitimacy of SGBs as regulators to a certain extent, yet concomitantly excludes absolute autonomy, imposes substantial limitations on their autonomy. García (146) argues that the CJEU limits the extent of that autonomy along two dimensions. First, the CJEU has acknowledged the necessity for SGBs to take into account the diverse interests of their stakeholders when formulating regulations and policies. Secondly, there is a necessity for a thorough examination of SGBs' regulations in the context of EU law. Autonomy of sport is the outcome of good governance, increased levels of transparency, effective accountability mechanisms, and enhanced stakeholder consideration. The autonomy of sport organisations is threatened by badly written regulations and the lack of effective enforcement of it, which opens the door to court cases.

Future research may focus on the evolving nature of the *lex sportiva*, in particular the balance between autonomy and accountability in global sport law, as well as the implications of increased legal scrutiny through state intervention and legal pluralism. It may explore how sport bodies can effectively navigate the legal landscape to protect their autonomy and the potential for reform to address governance failures. Engagement with EU institutions is needed to clarify the application of EU law to sport.

#### 3.2.4 What are limitations of the concept?

The limits and prerequisites to autonomy of sport result in different conceptualisations of the term in research, such as supervised (23), negotiated (5), pragmatic (21), responsible (85), earned (81), relative (97), and conditional (49) autonomy.

The limitation of the autonomy of sport concept is analysed through the issues surrounding the processes of organisational and institutional evolution of modern sport, in particular the governmentalisation and politicisation of sport (121, 69). Chappelet (3) maps out the key decisions in the evolution of “sports autonomy” (p. 21). Meier and García (13) suggest that the concept of sport autonomy has been heavily redefined by some of the very sport organisations that initially championed it. The creation of the World Anti-Doping Agency (WADA) in 1999, as a public-private partnership controlled and funded by governments and the Olympic Movement, illustrates how anti-doping efforts have challenged and eroded the idea of sport autonomy (13). Despite an implicit conviction that international sport cannot function properly if governments are allowed to interfere in its governance, the IOC itself recognises that it needs government support in certain areas. In a seminar on Autonomy of Olympic and Sport Movement in 2008 (122), principle number 7 of the IOC's Basic Universal Principles of Good Governance of the Olympic Movement was concluded stating that the sport movement and governments have “complementary missions”, and therefore emphasis was made on the importance of “cooperation, coordination, and consultation”. The records address the limitations of the concept of autonomy to grasp the realities of networked governance in the face of salient threats in the domain of global sport governance from which sport cannot protect itself alone (1). Namely: doping (55), match-fixing and illegal betting (123, 124), on-field corruption (125), corruption by sport governing body officials (104, 90, 126), corruption in major sporting events (127) abuse and harassment (128, 91), racism and other forms of discrimination (75, 91, 77, 129, 130), orginised crime (99, 131), hooliganism and spectator violence (98), third-party ownership of player (132). The authors repeatedly highlight that enhancing engagement and representation of stakeholders and installing a social dialogue among stakeholders is the counterstrategy to the threats outlined (82, 133, 50, 134).

Another set of records focused on the role and intervention of the intergovernmental organisations in limiting the autonomy of sport. Jedlicka (12) encourages an understanding of sport as a product of an international system and integrates international relations theory with sport governance research with the aim of enhancing the understanding of international sport's political status and impacts. The political autonomy of sport is ever-present in the records that explore how SGBs' use the principle of political neutrality to restrict athletes right to freedom of expression (70, 103, 135, 136). Other records aim to reconcile the principle of political neutrality with the Olympic movement's responses to the wars of aggression (18, 84, 137, 129). Waters (137) discusses the IOC's diplomatic efforts surrounding the dissolution of Yugoslavia and war in Bosnia-Herzegovina to preserve the autonomy of sport in the face of the United Nations Security Council (UNSC)'s use of sporting sanctions. In this respect, the IOC was forced into action by a binding request from the UNSC Resolution 757, which effectively limits the autonomy of sport. In this respect, we understand public authority exercised by an autonomous regulatory body – IOC – as *regulated* self-regulation because it takes place under the shadow of hierarchy (138).

The government's regulatory influence over the sport sector is evident in the establishment of governmental-affiliated bodies such as UK Sport, Sport Canada, Sport Australia, the Japan Sports Council, and the Sport and Recreation South Africa bodies. However, the concept of autonomy has increasingly become subject to interpretation by various governments. For example, in 2016, Sport England introduced the Code for Sports Governance, which stipulates mandatory compliance with the “gold standards of governance” that support strengthening SGBs' structures and policies but does not explicitly mention the notion of autonomy. According to the Governance Institute (139), “it is a bold move, representing definitively the end of autonomy, at least for funded organisations in the UK, and the introduction in its place of “earned autonomy”” (p. 5). The implementation of this framework necessitates the quantification of good governance in sport for SGBs to secure or uphold their funding. In this regard, Girginov (81) outlines the capacity of the governments to change the meaning of autonomy.

A financial dependency on a limited number of actors has the potential to engender a relationship of subordination, thereby restricting an organisation's autonomy and prompting activities that are not aligned with the sport organisation's vision and mission (140). Financial (over)dependency on government funding exposes organisations to external pressure, including the need to comply with government regulations, meet performance targets, and align with corporate social responsibility (CSR) practices (60). Moreover, funding agencies often exert influence through conditional financial support, where failure to meet expectations can result in sanctions or funding cuts. This, together with financial dependency on television rights and corporate sponsorship (92), can significantly shape organisational decision-making, as external stakeholders and “interested” government intervention play a growing role in setting strategic priorities and governance frameworks.

EU institutions, consisting of the Commission, Parliament, and Council of the EU (Committee of Ministers and Parliamentary Assembly), have been conditioning autonomy in sport on good governance since 2007. The Council of Europe underlines the importance of good governance in practice and warned that the autonomy of sport is earned rather than granted *ipso facto*. However, the EU institutions are not endorsing a specific set of good governance principles and monitoring its implementation. Thompson et al. (61) found 258 unique governance principles. The role of the EU institutions is of interest within the larger socio-political context (141). Hellmund (142) expands on the EU institutional structures outlining who the key actors in European sport policy are, what they do, and what influence they have in decision-making procedures (p. 27–33). While the European Parliament and the Council set the overall political direction and priorities, the European Commission exercises short-term authority by designing and implementing policies and decisions within that framework (143). Over the last five decades, there have been numerous activities of the EU institutions seeking to identify concepts such as the specificity and the autonomy of sport. Refer to the supplement file for the selected EU sport policy documents on autonomy of sport (see Supplementary Material 4B). Several authors (83, 67, 144) have pointed out the Olympic Movement's dilemma between its apolitical stance and governance responsibilities, with good governance serving as a “fig leaf” to justify engaging in political negotiation.

The records bring emphasis to the ratification of the Treaty of Lisbon in 2009 and how adoption of Article 165 of the Treaty on the Functioning of the European Union (TFEU) gave sport a constitutional footing and opened the political process by granting the EU a formal role in the field of sport. As such, “sporting bodies can no longer claim that sport is none of the EU's business” (49). Still, the Article 165 contains no clear reference to the autonomy of SGBs leaving some room for interpretation (145). García and Weatherill (49) conducted 45 semi-structured interviews with officials from EU institutions, national governments and sport organisations during the Treaty negotiations that revealed that SGBs have been able to exercise significant political leverage with the lobbying efforts. As the sporting landscape evolves, the rulings of the CJEU in *Superleague*, *Royal Antwerp* and *International Skating Union* can also be interpreted as a warning to the Commission, European Parliament, and Council of the EU on the limits of Article 165 TFEU in the development of a European sport policy (146).

A critical and interdisciplinary review of the autonomy of sport in the light of emerging developments seems warranted. As indicated by the Association of Summer Olympic International Federations (ASOIF) (147), there is a paucity of timely identification of the threats to the autonomy of sport. Such research should focus on a classification of the different developments (e.g., social, political, economic, technological, environmental) that may challenge the autonomy of sport. These factors have the potential to contribute to an erosion of sport autonomy and to threaten the integrity of sport – personal, competition, and institutional (148). More in-depths assessments of the current developments that affect the autonomy of sport could predict and decipher future changes, while the insights gained could be applied to the wider landscape of sport governance. A comprehensive autonomy disruption register could help derive recommendations on how SGBs could respond to selected challenges to their autonomy and remain resilient to the changing sport ecosystem.

## 4 Conclusion and limitations

The autonomy of sport is a longstanding principle of fundamental importance within the Olympic Movement and sport generally. Our results indicate that its importance is growing in light of certain developments (e.g., professionalisation, commercialisation, globalisation) in sport and its increasing interplays with geopolitics. Further, the exponential growth of scholarship in autonomy over the last two decades has been fuelled by the growing demand to describe and explain the evolving nature of autonomy of sport, and by normative concerns to promote ethically sound and managerially effective autonomy in the practice of international sport governance. While autonomy is often framed as a universal principle, its origins are Western European in cultural and geographical terms, shaping the way non-Europeans experience and govern sport. As the number of stakeholders in international sport governance continues to expand, autonomy finds itself at the centre of a complex (re)consideration of modernity. Recognising the great heterogeneity of SGBs, the autonomy of sport requires a sophisticated deconstruction of its multiple dimensions and conceptualisations in chosen contexts. Our scoping review contributed to this in many ways and provides important insights for policy-makers and researchers alike. Ultimately, synthesising and mapping the literature on autonomy of sport allows for a more comprehensive and nuanced understanding of how autonomy functions within international sport governance.

The limitation of scoping reviews (in contrast to systematic reviews) is that they attempt to provide extensive (rather than intensive) coverage of a particular subject matter (29). In our review we made the deliberate choice to remain at a general level in order to critically assess the concept. While particular reference is made to the evolving nature of the autonomy of selected SGBs, the intention is to rather remain at a macro- (or meso-) level and address the wider picture of the concept. At long last, our review is supposed to stimulate further critical research based on the future research directions offered.

## Data Availability

The original contributions presented in the study are included in the article/Supplementary Material, further inquiries can be directed to the corresponding author.

## Footnotes

^1^TI, title; AB, abstract; AW, keywords; SU, subject.

^2^This study uses the Council of Europe’s definition of sport, as proposed in its Revised European Sports Charter, which argues that: "Sport means all forms of physical activity, which through casual or organised participation, aim at expressing or improving physical fitness and mental well-being, forming social relationships or obtaining results in competition at all levels". [See Art. 2 of (39)].

^3^The IOC, together with FIFA and UEFA, can be considered the most powerful SGBs, both economically and politically, and the challenges to their autonomy have been of the most diverse nature.

## Supplementary material

Supplementary Material 1 Preferred Reporting Items for Systematic reviews and Meta-Analyses extension for Scoping Reviews (PRISMA-ScR) C.

Supplementary Material 2 Search details.

Supplementary Material 3 Title and abstract screener instructions.

Supplementary Material 4 List of included records.

Supplementary Material 5 Extraction guidance sheet for a systematic scoping review.

Supplementary Material 6 Summary of Extracted Data.

## References

[1] * Scheerder J. Conclusion: established models of European sport revisited from a socio-politological approach1. In: Porro NR Martelli S Testa A, editors. Sport, Welfare and Social Policy in the European Union. London: Routledge (2020). p. 153–68. Scopus. 10.4324/9781351118064-14

[2] * IOC. Olympic Rules (1949). Available at: https://stillmed.olympic.org/Documents/Olympic%20Charter/Olympic_Charter_through_time/1949-Olympic_Charter.pdf (Accessed April 7, 2025).

[3] * Chappelet J-L. Autonomy and governance: necessary bedfellows in the fight against corruption in sport. In: Transparency International, editor. Global Corruption Report Sport. 1st ed. London: Routledge (2016). p. 16–28. Available at: https://www.taylorfrancis.com/chapters/edit/10.4324/9781315695709-12/3-autonomy-governance-necessary-bedfellows-fight-corruption-sport-jean-loup-chappelet-idheap-swiss-graduate-school

[4] * IOC. IOC Code of Ethics (2024). Available at: https://stillmed.olympics.com/media/Document%20Library/OlympicOrg/Documents/Code-of-Ethics/Code-of-Ethics-ENG.pdf (Accessed April 7, 2025).

[5] * Chappelet J-L. Autonomy of Sport in Europe. Strasbourg: Council of Europe (2010).

[6] * IOC. IOC Olympic Charter (2025). Available at: https://stillmed.olympics.com/media/Documents/International-Olympic-Committee/IOC-Publications/EN-Olympic-Charter.pdf (Accessed April 7, 2025).

[7] * Zintz T Gérard S. Support the implementation of good governance in sport (SIGGS): a European project for national Olympic committees and national sport federations. In: Winand M Anagnostopoulos C, Research Handbook on Sport Governance. Cheltenham: Edward Elgar Publishing (2019). p. 53–71. 10.4337/9781786434821.00010

[8] Parrish R. The Autonomy of Sport: A Legal Analysis. 35th ed. Brussels: Sport and Citizenship (2016). p. 20–1. XVI. Available at: https://www.sportetcitoyennete.com/en/articles-en/the-autonomy-of-sport-a-legal-analysis

[9] Bach T. Statement on the occasion of the adoption of the resolution “Building a peaceful and better world through sport and the Olympic ideal” (2013). Available at: https://stillmed.olympic.org/Documents/IOC_President/2013-11-%206_Speech_IOC_President_Bach-%20Olympic_Truce_adoption_Speech_4_November.pdf (Accessed September 9, 2024).

[10] García B. UEFA And the European union: from confrontation to co-operation? J Contemp Eur Res. (2007) 3(3):202–23. 10.30950/jcer.v3i3.52

[11] IOC. Statement on the occasion of the adoption of the resolution “Building a peaceful and better world through sport and the Olympic ideal”, 68 Session of the UN General Assembly New York, 6 November 2013 (2013). Available at: https://stillmed.olympic.org/Documents/IOC_President/2013-11-6_Speech_IOC_President_Bach-Olympic_Truce_adoption_Speech_4_November.pdf (Accessed June 3, 2025).

[12] * Jedlicka SR. Sport governance as global governance: theoretical perspectives on sport in the international system. Int J Sport Pol Polit. (2018) 10(2):287–304. 10.1080/19406940.2017.1406974

[13] * Meier HE García B. Beyond sports autonomy: a case for collaborative sport governance approaches. Int J Sport Pol Polit. (2021) 13(3):501–16. 10.1080/19406940.2021.1905035

[14] * UNESCO. Final Report of the Intergovernmental Committee for Physical Education and Sport, 29–30 January 2015 (CIGEPS/2015/INF.REV) (2015). Available at: https://unesdoc.unesco.org/ark:/48223/pf0000232512 (Accessed February 24, 2025).

[15] * García B Meier HE Moustakas L. Racing to win: competition and co-operation between the national Olympic committee and public authorities in the development of the Botswana sport system. J South Afr Stud. (2023) 49(4):637–59. Scopus. 10.1080/03057070.2023.2289806

[16] * Geeraert A Alm J Groll M. Good governance in international sport organizations: an analysis of the 35 Olympic sport governing bodies. Int J Sport Pol Polit. (2013) 6(3):281–306. 10.1080/19406940.2013.825874

[17] * Allison L Tomlinson A. Understanding international sport organisations: principles, power and possibilities. In: Allison L Tomlinson A, editors. Understanding International Sport Organisations: Principles, Power and Possibilities. London: Routledge (2017). p. 244. 10.4324/9781315743875

[18] * Meeuwsen S Kreft L. Sport and politics in the twenty-first century. Sport Ethics Philos. (2023) 17(3):342–55. sph. 10.1080/17511321.2022.2152480

[19] * Schimank U. The autonomy of modern sport: dangerous and endangered. Eur J Sport Soc. (2005) 2(1):13–23. 10.1080/16138171.2005.11687762

[20] Chadwick S Anagnostopoulos C. A geopolitical economy of football: the case of Qatar. Int J Sport Pol Polit. (2023) 16(2):331–8. 10.1080/19406940.2023.2288815

[21] * Geeraert A Mrkonjic M Chappelet J-L. A rationalist perspective on the autonomy of international sport governing bodies: towards a pragmatic autonomy in the steering of sports. Int J Sport Pol Polit. (2015) 7(4):473–88. 10.1080/19406940.2014.925953

[22] Andersen JS. Sport is too loaded with conflicts of interests to clean up its own act. The 18th Council of Europe Conference of Ministers Responsible for Sport (2024). Available at: https://www.linkedin.com/company/play-the-game/posts/ (Accessed October 18, 2024).

[23] * Foster K. Can sport be regulated by Europe?: an analysis of alternative models. In: Caiger A Gardiner S, Professional Sport in the EU: Regulation and Re-regulation. The Hague: T.M.C. Asser Press (2000). p. 43–64. 10.1007/978-90-6704-455-4_4

[24] Dowling M Leopkey B Inoue Y Berg BK Smith L. Scoping reviews and structured research synthesis in sport: methods, protocol and lessons learnt. Int J Sport Pol Polit. (2020) 12(4):765–74. 10.1080/19406940.2020.1817126

[25] Forscher B. Chaos in the Brickyard. Science (1963) 142(3590):339. 10.1126/science.142.3590.339 17799464

[26] Tricco AC Lillie E Zarin W O’Brien KK Colquhoun H Levac D PRISMA Extension for scoping reviews (PRISMA-ScR): checklist and explanation. Ann Intern Med. (2018) 169(7):467–73. 10.7326/M18-0850 30178033

[27] Bevir M. The SAGE Handbook of Governance. Thousand Oaks, CA: SAGE Publications Ltd (2011). Available at: https://us.sagepub.com/en-us/nam/the-sage-handbook-of-governance/book233053

[28] Arksey H O’Malley L. Scoping studies: towards a methodological framework. Int J Soc Res Methodol. (2005) 8(1):19–32. 10.1080/1364557032000119616

[29] Levac D Colquhoun H O’Brien KK. Scoping studies: advancing the methodology. Implement Sci. (2010) 5(1):69. 10.1186/1748-5908-5-69 20854677 PMC2954944

[30] Munn Z Peters MDJ Stern C Tufanaru C McArthur A Aromataris E. Systematic review or scoping review? Guidance for authors when choosing between a systematic or scoping review approach. BMC Med Res Methodol. (2018) 18(1):143. 10.1186/s12874-018-0611-x 30453902 PMC6245623

[31] Peters MDJ Marnie C Tricco AC Pollock D Munn Z Alexander L Updated methodological guidance for the conduct of scoping reviews. JBI Evid Synth. (2020) 18(10):2119–26. 10.11124/JBIES-20-00167 33038124

[32] * Shilbury D Ferkins L. In: Shilbury D Ferkins L, editors. Routledge Handbook of Sport Governance. 1st ed. London: Routledge (2019). p. 3–17. 10.4324/9780429440250

[33] Constandt B Vertommen T Cox L Kavanagh E Kumar BP Pankowiak A Quid interpersonal violence in the sport integrity literature? A scoping review. Sport Soc. (2024) 27(1):162–80. 10.1080/17430437.2023.2233433

[34] Dowling M Leopkey B Smith L. Governance in sport: a scoping review. J Sport Manage. (2018) 32(5):438–51. 10.1123/jsm.2018-0032

[35] Inoue Y Berg BK Chelladurai P. Spectator sport and population health: a scoping study. J Sport Manage. (2015) 29(6):705–25. 10.1123/JSM.2014-0283

[36] Vanwersch L Willem A Constandt B Hardyns W. A scoping review of the causes and consequences of fraud in sport. J Sport Soc Issues. (2022) 46(6):546–84. 10.1177/01937235221119811

[37] Peters MDJ Godfrey CM Khalil H McInerney P Parker D Soares CB. Guidance for conducting systematic scoping reviews. Int J Evid Based Healthc. (2015) 13(3):141–6. 10.1097/XEB.0000000000000050 26134548

[38] OSF. The autonomy of sport concept: A systematic scoping review. OSF Registries (2024). Available at: https://osf.io/apk4h/?view_only=3c8225f934c64014b1d349340da39e78 (Accessed June 3, 2025).

[39] * Council of Europe. Revised European Sports Charter. Strasbourg: Council of Europe (1992). Available at: https://edoc.coe.int/en/sport-for-all/11299-revised-european-sports-charter.html?utm_source=chatgpt.com (Accessed October 18, 2024).

[40] Mazzucco M. Lex Sportiva: Sports Law as a Transnational Autonomous Legal Order. Canada University of Victoria (2010). Available at: https://www.academia.edu/436095/Lex_Sportiva_Sports_Law_as_a_Transnational_Autonomous_Legal_Order (Accessed June 3, 2025).

[41] Adams RJ Smart P Huff AS. Shades of grey: guidelines for working with the grey literature in systematic reviews for management and organizational studies. Int J Manage Rev. (2017) 19(4):432–54. 10.1111/ijmr.12102

[42] Godin K Stapleton J Kirkpatrick SI Hanning RM Leatherdale ST. Applying systematic review search methods to the grey literature: a case study examining guidelines for school-based breakfast programs in Canada. Syst Rev. (2015) 4(1):138. 10.1186/s13643-015-0125-0 26494010 PMC4619264

[43] McHugh ML. Interrater reliability: the kappa statistic. Biochem Med (Zagreb). (2012) 22:276–82. 10.11613/BM.2012.031 23092060 PMC3900052

[44] Pollock D Peters MDJ Khalil H McInerney P Alexander L Tricco AC Recommendations for the extraction, analysis, and presentation of results in scoping reviews. JBI Evid Synth. (2023) 21(3):520–32. 10.11124/JBIES-22-00123 36081365

[45] Ghalibaf AK Nazari E Gholian-Aval M Tabesh H Tara M. Comprehensive overview of computer-based health information tailoring: a scoping review protocol. BMJ Open. (2017) 7(12):e019215. 10.1136/bmjopen-2017-019215 29284722 PMC5770833

[46] * García B Meier HE. The “autonomy” of developing countries in the Olympic movement: assessing the fate of sports governance transplants in the global south. Front Sports Act Living. (2022) 4:972717. 10.3389/fspor.2022.972717 36532104 PMC9751918

[47] * Fischer P Kornbeck J Miège C Stopper M. Responsible sport and state oversight: sports organisations as civil society organisations and private regulators in France and Germany. Int Sports Law J. (2023) 24:3–19. Scopus. 10.1007/s40318-023-00252-7

[48] * Choi S. Enhancement Plan to Increase Self-revenue for the Korean Sport & Olympic Committee, Securing and Strengthening Autonomy Focusing on Analysis of NOC’s Financial Statement. Ottawa: Olympic World Library (2023).

[49] * García B Weatherill S. Engaging with the EU in order to minimize its impact: sport and the negotiation of the treaty of Lisbon. J Eur Public Policy. (2012) 19(2):238–56. Scopus. 10.1080/13501763.2011.609710

[50] * Geeraert A. New EU governance modes in professional sport: enhancing throughput legitimacy. J Contemp Eur Res. (2014) 10(3):302–21. 10.30950/jcer.v10i3.562

[51] * Geeraert A. The limits and opportunities of self-regulation: achieving international sport federations’ compliance with good governance standards. Eur Sport Manage Q. (2019) 19(4):520–38. 10.1080/16184742.2018.1549577

[52] * Minikin B. Legitimacy and democracy: implications for governance in sport. Sport Bus Manage Int J. (2015) 5(5):435–50. Scopus. 10.1108/SBM-03-2015-0010

[53] * Winand M Steen A Kasale LL. Performance management practices in the sport sector: an examination of 32 Scottish national sport organizations. J Global Sport Manage. (2023) 8(4):739–62. sph. 10.1080/24704067.2021.1899765

[54] * Abrutyn S. “Integrity, sportsmanship, character”: baseball’s moral entrepreneurs and the production and reproduction of institutional autonomy. Sociol Q. (2018) 59(3):519–44. 10.1080/00380253.2018.1479203

[55] * Harris S Dowling M Houlihan B. An analysis of governance failure and power dynamics in international sport: the Russian doping scandal. Int J Sport Pol Polit. (2021) 13(3):359–78. sph. 10.1080/19406940.2021.1898443

[56] * Szatkowski M. Analysis of the sports model in selected western European countries. J Phys Educ Sport. (2022) 22(3):829–39. 10.7752/jpes.2022.03105

[57] * Wickstrøm M Alvad S. Autonomy in National Olympic Committees 2017. An autonomy index. Play the Game (2017).

[58] * Yaghi A Almutawwa R. Perceptions of sport governance and performance in United Arab Emirates. Public Organ Rev. (2023) 23(1):113–31. Scopus. 10.1007/s11115-022-00631-y

[59] * Ioannidis G. Football intermediaries and self-regulation: the need for greater transparency through disciplinary law, sanctioning and qualifying criteria. Int Sports Law J. (2019) 19(3):154–70. 10.1007/s40318-019-00159-2

[60] * Zeimers G Lefebvre A Winand M Anagnostopoulos C Zintz T Willem A. Organisational factors for corporate social responsibility implementation in sport federations: a qualitative comparative analysis. Eur Sport Manage Q. (2021) 21(2):173–93. 10.1080/16184742.2020.1731838

[61] Thompson A Lachance EL Parent MM Hoye R. A systematic review of governance principles in sport. Eur Sport Manage Q. (2023) 23(6):1863–88. 10.1080/16184742.2022.2077795

[62] * Budevici-Puiu L Manolachi V. The autonomy and specifity of sport in a national and European context. Rev Romaneasca Pentr Educ Multidimens. (2022) 14(3):457–65. 10.18662/rrem/14.3/619

[63] * Geeraert A. National Sports Governance Observer. Final report. Play the Game (2018). Available at: https://www.dshs-koeln.de/fileadmin/redaktion/user_upload/National_Sports_Governance_Observer_-_final_report.pdf (Accessed June 3, 2025).

[64] * Budevici-Puiu L Manolachi V Manolachi V. Specific elements of good governance in sport, as important factors in ensuring the management. Rev Romaneasca Pentr Educ Multidimens. (2020) 12(4):328–37. 10.18662/rrem/12.4/348

[65] * Scheerder J Claes E Willem A. Does it take two to tango? The position and power of national sport bodies compared to their public authorities. In: Scheerder J Willem A Claes E, editors. Sport Policy Systems and Sport Federations: A Cross-National Perspective. London: Palgrave Macmillan (2017). p. 1–17. Scopus. 10.1057/978-1-137-60222-0_1

[66] * Dolbysheva N. Historical features of the development of the autonomy of non-Olympic sports in the system of the international sports movement in the period of modern and contemporary history. Sport I Turystyka. (2022) 5(3):11–30. Scopus. 10.16926/sit.2022.03.01

[67] * Girginov V. A cultural perspective on good governance in sport. In: Research Handbook on Sport Governance. Cheltenham: Edward Elgar Publishing (2019). p. 89–101.

[68] * Panagiotopoulos DP Kallimani Z. Appointment of temporary administration in the hellenic football federation Greek government—FIFA intervention. Rassegna Diritto Economia Dello Sport. (2017) 12(2):543–9. https://search-ebscohost-com.kuleuven.e-bronnen.be/login.aspx?direct=true&db=sph&AN=138419692&site=ehost-live&scope=site (Accessed June 06, 2025).

[69] * van der Walt JC. Autonomy in sport and South African statutory law: a critical evaluation. S Afr J Res Sport Phys Educ Recreation. (1982) 5(1):91–103. sph.

[70] * Abanazir C. Political Expression in Sport: Transnational Challenges, Moral Defences. London: Routledge (2022). p. 193. Scopus. 10.4324/9781003241102

[71] * Baddeley M. The extraordinary autonomy of sports bodies under Swiss law: lessons to be drawn. Int Sports Law J. (2020) 20(1–2):3–17. 10.1007/s40318-019-00163-6

[72] * Bruyninckx H. Sports governance: between the obsession with rules and regulation and the aversion to being ruled and regulated. In: Segaert B Theeboom M Timmerman C Vanreusel B, editors. Sports Governance, Development and Corporate Responsibility. London: Routledge (2012). p. 107–21. Available at: https://api.taylorfrancis.com/content/chapters/edit/download?identifierName=doi&identifierValue=10.4324/9780203106020-10&type=chapterpdf

[73] * Coleman DL. Symposium on the olympics and international law the Olympic movement in international law. AJIL Unbound. (2020) 114:385–90. 10.1017/aju.2020.75

[74] * Duval A. Transnational sports law: the living lex sportiva. In: Zumbansen P, editor. The Oxford Handbook of Transnational Law. Oxford: Oxford Academic (2021). p. 493–512. Scopus. 10.1093/oxfordhb/9780197547410.013.23

[75] * González CP. The effective application of international human rights law standards to the sporting domain: should UN monitoring bodies take central stage? Int Sports Law J. (2022) 22(2):152–64. Scopus. 10.1007/s40318-021-00209-8

[76] * Kornbeck J. EU Antitrust Law and Sport Governance: The Next Frontier? 1st ed. London: Routledge (2022). 10.4324/9781003305989

[77] * Lenskyj HJ. Gender, Athletes’ Rights, and the Court of Arbitration for Sport. Amsterdam: Emarald Publishing (2018). p. 1.

[78] * Lewandowski W. The implications of the recent jurisprudence of the court of justice of the European union for the protection of the fundamental rights of athletes and the regulatory autonomy of sporting federations. Tilburg Law Rev. (2020) 25(1):55–66. 10.5334/TILR.193

[79] * Vieweg K. The legal autonomy of sport organisations and the restrictions of European law. In: Caiger A Gardiner S, editors. Professional Sport in the EU: Regulation and Re-regulation. The Hague: Springer (2000). p. 83–106.

[80] * Weatherill S. Principles and Practice in EU Sports law. London: Oxford University Press (2017).

[81] * Girginov V. The numbers game: quantifying good governance in sport. Eur Sport Manage Q. (2023) 23(6):1889–905. 10.1080/16184742.2022.2078851

[82] Colucci M Geeraert A. The ’Social dialogue’ in European professional football (2013).

[83] * Chappelet J-L Mrkonjic M. Assessing sport governance principles and indicators. In: Research Handbook on Sport Governance. Cheltenham: Edward Elgar Publishing (2019). p. 10–28. Available at: https://www.elgaronline.com/abstract/edcoll/9781786434814/9781786434814.00008.xml

[84] * Næss HE. The Neutrality Paradox in Sport: Governance, Politics and Human Rights After Ukraine. Cham: Springer International Publishing (2022). 10.1007/978-3-031-15680-9

[85] * Rook W Prado T Heerdt D. Responsible sport: no going back. Int Sports Law J. (2023) 23(1):85–98. Scopus. 10.1007/s40318-022-00231-4

[86] Geeraert A. A rational choice perspective on good governance in sport: the necessity of rules of the game. In: Geeraert A van Eekeren F, editors. Good Governance in Sport. London: Routledge (2021). p. 15–29.

[87] Gathii JT. TWAIL: A Brief History of its Origins, its Decentralized Network, and a Tentative Bibliography (SSRN Scholarly Paper 1933766). Amsterdam: Social Science Research Network (2011). Available at: https://papers.ssrn.com/abstract=1933766

[88] * Foster K. Is there a global sports law? In: Siekmann RCR Soek J, Lex Sportiva: What is Sports Law? The Hague: T. M. C. Asser Press (2012). p. 35–52. 10.1007/978-90-6704-829-3_2

[89] Watson A. Legal Transplants: An Approach to Comparative law. Athens: Scottish Academic Press (1974).

[90] * Hylton JG. How FIFA used the principle of autonomy of sport to shield corruption in the sepp blatter era. Md J Int’l L. (2017) 32:134. Available at: https://digitalcommons.law.umaryland.edu/mjil/vol32/iss1/6

[91] * Hessert B. The protection of minor athletes in sports investigation proceedings. Int Sports Law J. (2021) 21(1–2):62–73. Scopus. 10.1007/s40318-020-00177-5

[92] * Wenn SR. Financing the Olympic Movement—Early Developments and Evolutions. Laussane: The Olympic Studies Centre (2024).

[93] * Ponkina AI. Autonomy of sport: legal aspects. Int Sports Law Rev Pandektis. (2013) 10(1/2):204–15. sph.

[94] * Lewis A Taylor J. Sport: Law and Practice. London: Bloomsbury Publishing (2021).

[95] * Court of Justice of the European Union. European Superleague Company, SL v Fédération Internationale de Football Association (FIFA) and Union of European Football Associations (UEFA), Case C-333/21. EUR-Lex (2023). Available at: https://eur-lex.europa.eu/legal-content/en/TXT/?uri=CELEX:62021CJ0333 (Accessed February 28, 2025).

[96] * Court of Justice of the European Union. International Skating Union v European Commission, Case C-124/21 P. EUR-Lex (2023). Available at: https://eur-lex.europa.eu/legal-content/EN/TXT/?uri=CELEX%3A62021CJ0124 (Accessed February 28, 2025).

[97] * Pearson G. Sporting justifications under EU free movement and competition law: the case of the football ‘transfer system’. Eur Law J. (2015) 21(2):220–38. Scopus. 10.1111/eulj.12110

[98] Council of Europe. European Convention on an Integrated Safety, Security and Service Approach at Football Matches and Other Sport Events (1985). Available at: https://rm.coe.int/168007a086 (Accessed February 24, 2025).

[99] * UN. Convention Against Transnational Organized Crime (2003). Available at: https://www.unodc.org/unodc/en/organized-crime/intro/UNTOC.html (Accessed February 24, 2025).

[100] * UNESCO. International Convention against Doping in Sport (2005). Available at: https://www.wada-ama.org/en/resources/unesco-international-convention-against-doping-sport (Accessed February 24, 2025).

[101] * Council of Europe. The Convention on the Manipulation of Sport Competitions (the Macolin Convention) (2014). Available at: https://rm.coe.int/16801cdd7esport (Accessed February 24, 2025).

[102] Duval A. Embedded lex sportiva: the Swiss roots of transnational sports law and governance. In: Duval A Krüger A Lindholm J, editors. The European Roots of the lex Sportiva: How Europe Rules Global Sport. London: Hart Publishing Ltd (2024). p. 17–40.

[103] * Di Marco A. Athletes’ freedom of expression: the relative political neutrality of sport. Hum Rights Law Rev. (2021a) 21(3):620–40. 10.1093/hrlr/ngab009

[104] * Di Marco A. Sports economy and fight against corruption: which limits to the sporting organisations autonomy? Eur Bus Law Rev. (2021b) 32(5):877–904. 10.54648/EULR2021031

[105] * Hoye R. Sport governance. In: Henry EB Ko L-M, editors. Routledge Handbook of Sport Policy. London: Routledge (2013). p. 331–40.

[106] * Chappelet J-L. Beyond governance: the need to improve the regulation of international sport. Sport Soc. (2017) 21(5):724–34. 10.1080/17430437.2018.1401355

[107] * Weatherill S. Saving football from itself: why and how to Re-make EU sports law. Camb Yearb Eur Legal Stud. (2022) 24:4–23. Scopus. 10.1017/cel.2022.3

[108] * Foster K. The juridification of sport. In: Greenfield S Osborn G, editors. Readings in law and Popular Culture. London: Routledge (2007). p. 163–90. Available at: https://www.taylorfrancis.com/chapters/edit/10.4324/9780203963838-14/juridification-sport-ken-foster

[109] * Parrish R. Lex sportiva and EU sports law. Eur Law Rev. (2012) 37(6):716–33.

[110] * Vieweg K. Lex sportiva and the fairness principle. Int Sports Law Rev Pandektis. (2014) 10(3/4):382–94. sph.

[111] * Foster K. Global sports law revisited. Ent Sports Law J. (2019) 17(2–4):1–14. sph. 10.16997/eslj.228

[112] Latty F. La lex Sportiva: Recherche sur le Droit Transnational. Martinus Nijhoff Publishers (2007).

[113] Duval A. Lex sportiva: a playground for transnational law. Eur Law J. (2013) 19(6):822–42. 10.1111/eulj.12067

[114] Coccia M Colucci M, editors. International Sports Justice. Sports Law and Policy Centre (2024).

[115] * Duval A Van Rompuy B. Protecting athletes’ right to a fair trial through EU competition law: the pechstein case. In: Paulussen C Takacs T Lazić V Rompuy BV, editors. Fundamental Rights in International and European Law: Public and Private Law Perspectives. London: Routledge (2015). p. 245–78. Scopus. 10.1007/978-94-6265-088-6_11

[116] * Di Marco A. Human rights in the Olympic movement: the application of international and European standards to the lex sportiva. Neth Q Hum Rights. (2022) 40(3):244–68. 10.1177/09240519221112554

[117] * Court of Justice of the European Union. Advocate General’s Opinion in Case C-600/23: Royal Football Club Seraing (2025). Available at: https://curia.europa.eu/jcms/upload/docs/application/pdf/2025-01/cp250006en.pdf (Accessed February 28, 2025).

[118] * Foster K. Alternative models for the regulation of global sport. In: Allison L, editor. The Global Politics of Sport. London: Routledge (2004). p. 57–78.

[119] * Parrish R. Judicial intervention and sporting autonomy: defining the territories of European union involvement in sport. Eur Sport Manage Q. (2002) 2(4):296–307. 10.1080/16184740208721930

[120] James M Duval A. Another bosman moment? The decisions of the court of justice of the European union on 21 December 2023 and the future of transnational sports governance. Int Sports Law J. (2023) 23(4):405–8. 10.1007/s40318-024-00268-7

[121] * Porat AB. Football ‘made in Israel’. Isr Stud Rev. (2019) 34(3):1–16. Scopus. 10.3167/isr.2019.340302

[122] * IOC. Basic Universal Principles of Good Governance of the Olympic and Sports Movement. Seminar on Autonomy of Olympic and Sport Movement, 11–12 February 2008 (2008). Available at: https://stillmed.olympic.org/Documents/Conferences_Forums_and_Events/2008_seminar_autonomy/Basic_Universal_Principles_of_Good_Governance.pdf (Accessed April 7, 2025).

[123] * Mravec L. Match-fixing as a threat to sport: ethical and legal perspectives. Stud Sport. (2021) 15(2):37–48. Scopus. 10.5817/STS2021-2-4

[124] * Serby T. The council of Europe convention on manipulation of sports competitions: the best bet for the global fight against match-fixing? Int Sports Law J. (2015) 15(1–2):83–100. Scopus. 10.1007/s40318-015-0069-5

[125] * Serby T. Sports corruption: sporting autonomy, lex sportiva and the rule of law. Ent Sports Law J. (2017) 15(1):1–9. sph. 10.16997/eslj.204

[126] * Kruessmann T. Extending integrity to third parties: in search of a new model for anti-corruption in sports. Int Sports Law J. (2019) 18(3–4):136–49. Scopus. 10.1007/s40318-018-0137-8

[127] * UNODC. SAFEGUARDING SPORT FROM CORRUPTION. Towards effective Implementation of resolution 7/8 on corruption in sport (2019).

[128] * Donnelly P Kerr G Kidd B. Contesting the autonomy of sport to realize the right to safe sport: a Canadian case study. Int Sports Law J. (2022) 22(2):165–70. 10.1007/s40318-022-00225-2

[129] * Wiater P. Chaos in the sporting world over Russia’s war of aggression: political neutrality in light of human rights protection. Bus Hum Rights J. (2023) 8(3):461–7. Scopus. 10.1017/bhj.2023.32

[130] * UN. Guiding Principles on Business and Human Rights: Implementing the United Nations “Protect, Respect and Remedy” Framework (2011). Available at: https://www.ohchr.org/en/issues/business/pages/businessindex.aspx (Accessed February 24, 2025).

[131] * UNODC. Global Report on Corrupion in Sport (2021).

[132] * Flanagan CA. The corridor of uncertainty: part two, why attempts to regulate the financial aspects of football are met with legal challenges. Int Sports Law J. (2018) 18(1–2):29–38. Scopus. 10.1007/s40318-018-0125-z

[133] * García B. From regulation to governance and representation: agenda-setting and the EU’s involvement in sport. Ent Sports Law J. (2016) 5:1. 10.16997/eslj.73

[134] * Parrish R McArdle D. Beyond bosman: the European union’s influence upon professional Athletes’ freedom of movement. Sport Soc. (2004) 7(3):403–19. sph. 10.1080/1743043042000291712

[135] * Modi T. To what extent is rule 50 of the Olympic charter valid? Balancing athletes freedom of expression and the mythical political neutrality of sport. Int Sports Law J. (2023) 23(3):368–89. Scopus. 10.1007/s40318-023-00249-2

[136] * Schwab B. ‘Celebrate humanity’: reconciling sport and human rights through athlete activism. J Legal Aspects Sport. (2018) 28(2):170–207. sph. 10.18060/22570

[137] * Waters L. In pursuit of prestige: the international Olympic committee’s peace efforts in bosnia-Herzegovina (1992–1994). Int J History Sport. (2023) 40(15):1347–63. sph. 10.1080/09523367.2024.2312153

[138] Coni-Zimmer M Wolf KD Collin P. Editorial to the issue on legitimization of private and public regulation: past and present. Politics Gov. (2017) 5(1):1–5. 10.17645/pag.v5i1.915

[139] The Governance Institute. The future of sports governance—Beyond autonomy. ICSA (2019). Available at: https://www.sportsgovernanceacademy.org.uk/media/axdbqjo2/the-future-of-sports-governance-beyond-autonomy.pdf (Accessed October 29, 2024).

[140] * SIGGS. Roadmap Principle 2: Autonomy & Accountability (2019).

[141] * Halleux V. EU sport policy: An overview (2015). Available at: https://policycommons.net/artifacts/1336010/eu-sport-policy/1942855/ (Accessed June 06, 2025).

[142] Hellmund. Guide to EU Sport Policy. EOC EU Office (2017).

[143] * Alm J. Accountability and good governance. In: Alm J, editor. Action for Good Governance in International Sports Organisations. Denmark: Play the Game (2013). p. 22–5. Available at: https://www.sport.ee/et/file/d0ad60723aeaad8b176d37aa0c364695/taani_2013_final_report_aggis_full_version.pdf#page=22

[144] * Næss HE. The normative legitimacy gap: international sports associations, human rights and stakeholder democracy. Sport Ethics Phil. (2020) 14(2):129–45. sph. 10.1080/17511321.2019.1566272

[145] * Weatherill S. EU Sports law: the effect of the Lisbon treaty. In: Biondi A Eeckhout P Ripley S, editors. EU Law After Lisbon. Oxford: Oxford Academic (2012). p. 403–20. Scopus. 10.1093/acprof:oso/9780199644322.003.0019

[146] * García B. Down with the politics, up with the law! Reinforcing EU law’s supervision of sport autonomy in Europe. Int Sports Law J. (2024) 23(4):416–21. 10.1007/s40318-024-00264-x

[147] ASOIF. Future of Global Sport (2019). Available at: https://www.asoif.com/sites/default/files/download/future_of_global_sport.pdf (Accessed April 7, 2025).

[148] Cleret L McNamee M Page S. ’Sports integrity’ needs sports ethics (and sports philosophers and sports ethicists too). Sport Ethics Phil. (2015) 9(1):1–5. 10.1080/17511321.2015.1049015

[149] Stevenson M Taylor BJ Knox J. Risk in dementia care: searching for the evidence. Health Risk Soc. (2015) 18(1–2):4–20. 10.1080/13698575.2015.1119256

