# Supplementary material for: The autonomy of sport concept: a scoping review
Source: Front Sports Act Living. 2025 Jun 19;7:1593673. doi: 10.3389/fspor.2025.1593673 (PMC12222117; doi:10.3389/fspor.2025.1593673)
Supplement: Supplementary Material 2 — Search details. [file Table2.docx]

Supplementary Material 2: Search details

1. Electronic databases and search engine

| **Database/platform:** | **Scopus (Elsevier)** |
| --- | --- |
| **Date of search:** | May 22, 2024 |
| **Search query:** | TITLE-ABS-KEY (autonomy OR self-regulation OR self-review OR self-governance OR freedom OR independence OR steering ) AND TITLE-ABS-KEY ( sport* OR "sport* organi*" OR "sport* governing bod*" OR "sport* federation*" OR "sport* association*") AND ( LIMIT-TO (LANGUAGE, "English") |
| **Number of hits:** | 4.978 |

| **Database/platform:** | **SPORTDiscus (EBSCOhost)** |
| --- | --- |
| **Date of search:** | May 22, 2024 |
| **Search query:** | ((TI autonomy OR self-regulation OR self-review OR self-governance OR freedom OR independence OR steering) OR (AB autonomy OR self-regulation OR self-review OR self-governance OR freedom OR independence OR steering) OR (KW autonomy OR self-regulation OR self-review OR self-governance OR freedom OR independence OR steering) OR (SU autonomy OR self-regulation OR self-review OR self-governance OR freedom OR independence OR steering) ) AND ( (TI sport* OR "sport* organi*" OR "sport* governing bod*"OR "sport* federation*" OR "sport* association*") OR (AB sport* OR "sport* organi*" OR "sport* governing bod*"OR "sport* federation*" OR "sport* association*") OR (KW sport* OR "sport* organi*" OR "sport* governing bod*"OR "sport* federation*" OR "sport* association*") OR (SU sport* OR "sport* organi*" OR "sport* governing bod*" OR "sport* federation*" OR "sport* association*"))  TI = title, AB = abstract, AW = keywords, SU = subject |
| **Number of hits:** | 3.224 |

| **Database/platform:** | **Web of Science (Web of Science Core Collection).** |
| --- | --- |
| **Date of search:** | May 22, 2024 |
| **Search query:** | ALL=((TI autonomy OR self-regulation OR self-review OR self-governance OR freedom OR independence OR steering) OR (AB autonomy OR self-regulation OR self-review OR self-governance OR freedom OR independence OR steering) OR (AK autonomy OR self-regulation OR self-review OR self-governance OR freedom OR independence OR steering) OR (SU autonomy OR self-regulation OR self-review OR self-governance OR freedom OR independence OR steering) )) AND ALL=((TI sport* OR "sport* organi*" OR "sport* governing bod*"OR "sport* federation*" OR "sport* association*") OR (AB sport* OR "sport* organi*" OR "sport* governing bod*"OR "sport* federation*" OR "sport* association*") OR (AK sport* OR "sport* organi*" OR "sport* governing bod*"OR "sport* federation*" OR "sport* association*") OR (SU sport* OR "sport* organi*" OR "sport* governing bod*" OR "sport* federation*" OR "sport* association*"))  TI = title, AB = abstract, AK = keywords, SU = subject |
| **Number of hits:** | 645 |

| **Database/platform:** | **Google Scholar** |
| --- | --- |
| **Library:** | Free access |
| **Date of search:** | May 24, 2024 |
| **Limits:** | In: "All fields"  Date range: All years |
| **Search query:** | (autonomy OR self-regulation OR self-review OR self-governance OR freedom OR independence OR steering) AND (sport* OR "sport* organi*" OR "sport* governing bod*" OR "sport* federation*" OR "sport* association*") |
| **Number of hits:** | 1. (the first thirty pages of the search’s hits) |

1. **Web search and websites**

| **Website:** | **International Olympic Committee (IOC)** |
| --- | --- |
| **Date of search:** | October 4, 2024 |
| **URL:** | https://olympics.com/ioc |
| **Number of hits:** | 73 |

| **Website:** | **Association of Summer Olympic International Federations (ASOIF)** |
| --- | --- |
| **Date of search:** | October 4, 2024 |
| **URL:** | https://www.asoif.com/ |
| **Number of hits:** | 10 |

| **Website:** | **Association of International Olympic Winter Sports Federations (AIOWF)** |
| --- | --- |
| **Date of search:** | October 4, 2024 |
| **URL:** | https://olympics.com/ioc/international-federations/wof |
| **Number of hits:** | 4 |

| **Website:** | **Fédération Internationale de Football Association (FIFA)** |
| --- | --- |
| **Date of search:** | October 4, 2024 |
| **URL:** | https://www.fifa.com/de |
| **Number of hits:** | 49 |

| **Website:** | **Union of European Football Associations (UEFA)** |
| --- | --- |
| **Date of search:** | October 4, 2024 |
| **URL:** | https://www.uefa.com/ |
| **Number of hits:** | 13 |

| **Database/platform:** | **Council of Europe** |
| --- | --- |
| **Date of search:** | October 4, 2024 |
| **URL:** | https://www.coe.int/en/web/documents-records-archives-information/search1 |
| **Number of hits:** | 6.619 |
| **Notes:** | Reviewed the first thirty pages of the search’s hits |

| **Database/platform:** | **European Parliament** |
| --- | --- |
| **Date of search:** | October 4, 2024 |
| **URL:** | https://www.europarl.europa.eu/portal/en |
| **Number of hits:** | 1.417 |
| **Notes:** | Reviewed the first thirty pages of the search’s hits |

| **Database/platform:** | **European Commission** |
| --- | --- |
| **Date of search:** | October 18, 2024 |
| **URL:** | https://eur-lex.europa.eu/homepage.html?lang=en |
| **Number of hits:** | 2.040 |
| **Notes:** | Reviewed the first thirty pages of the search’s hits |

| **Website:** | **International Partnership against Corruption in Sport (IPACS)** |
| --- | --- |
| **Date of search:** | October 4, 2024 |
| **URL:** | https://www.ipacs.sport/ |
| **Number of hits:** | 2 |

| **Database/platform:** | **United Nations Office on Drugs and Crime (UNODC)** |
| --- | --- |
| **Date of search:** | October 7, 2024 |
| **URL:** | https://www.unodc.org/unodc/index.html |
| **Number of hits:** | 269 |

| **Website:** | **United Nations Educational, Scientific and Cultural Organization (UNESCO)** |
| --- | --- |
| **Date of search:** | October 7, 2024 |
| **URL:** | https://unesdoc.unesco.org/home |
| **Number of hits:** | 4.187 |
| **Notes:** | Reviewed the first thirty pages of the search’s hits |

| **Database/platform:** | **Olympic World Library** |
| --- | --- |
| **Date of search:** | October 7, 2024 |
| **URL:** | https://library.olympics.com/accueil.aspx |
| **Number of hits:** | 33 |

| **Database/platform:** | **Open Grey** |
| --- | --- |
| **Date of search:** | October 7, 2024 |
| **URL:** | https://opengrey.eu/ |
| **Number of hits:** | 12 |

| **Database/platform:** | **Court of Arbitration for Sports** |
| --- | --- |
| **Date of search:** | October 9, 2024 |
| **URL:** | https://jurisprudence.tas-cas.org/Search/results.aspx#k= |
| **Number of hits:** | “autonomy” AND (self-regulation OR self-review OR self-governance OR independence OR freedom OR steering) [164]  *Keywords* (predetermined by CAS):   - Autonomy of the association [48] - Autonomy of the association (limitations) [12] - Autonomy to determine sanctions [24] |

| **Database/platform:** | **Implementation of Good Governance in Sport (SIGGS)** |
| --- | --- |
| **Date of search:** | October 9, 2024 |
| **URL:** | https://www.siggs.eu/index.html |
| **Number of hits:** | 2 |

| **Database/platform:** | **Play the Game/Sports Governance Observer (SGO)** |
| --- | --- |
| **Date of search:** | October 9, 2024 |
| **URL:** | https://www.playthegame.org/projects/sports-governance-observer-sgo/ |
| **Number of hits:** | 9 |
