# Supplementary material for: The autonomy of sport concept: a scoping review
Source: Front Sports Act Living. 2025 Jun 19;7:1593673. doi: 10.3389/fspor.2025.1593673 (PMC12222117; doi:10.3389/fspor.2025.1593673)
Supplement: Supplementary Material 3 — Title and abstract screener instructions. [file Table3.docx]

Supplementary Material 3: Title and abstract screener instructions

**Screener instructions:**

1.     Use Google Chrome to sign in the Rayyan with the corporate email address.

2.     Ensure “blind on mode” is turned on through the whole period of the screening.

3.     Screen the title and abstract in consistent order.

4.     Identify as:

Include if the record mentions the autonomy/self-regulation/self-review/self-governance/independence/freedom/steering and sport organisation/governing body/association/federation in abstract, keywords and title; in English

Exclude if the record does not fall into the scope (non-sport texts) and potentially does not answer the research questions (not focuses on the governance of sport, organised sport); no mention of autonomy/self-regulation/self-review/self-governance/independence/freedom/steering and sport organization/governing body/association/federation in abstract, keywords and title; not in English;

Maybe if the record does not provide enough information to assess.

**Screening reliability:**

To calculate the Cohen’s Kappa coefficient for inter-rater reliability, the «blind off» mode will be set in the Rayyan. Also, having «blind off» will give the possibility to see «in conflict» records.

**Screening reconciliation procedure:**

In the instances whereby a record would be identified for inclusion by one screener and not another, the record will be subjected to a full-text screening.
