# Supplementary material for: The autonomy of sport concept: a scoping review
Source: Front Sports Act Living. 2025 Jun 19;7:1593673. doi: 10.3389/fspor.2025.1593673 (PMC12222117; doi:10.3389/fspor.2025.1593673)
Supplement: Supplementary Material 4 — List of included records. [file Table4.docx]

Supplementary Material 4

A. List of included records from electronic databases

1. Abanazir, C. (2022). Political Expression in Sport: Transnational Challenges, Moral Defences. *Political Expr. in Sport: Transnational Challenges, Moral Defences*, 1.
2. Abrutyn, S. (2018). “Integrity, Sportsmanship, Character”: Baseball’s Moral Entrepreneurs and the Production and Reproduction of Institutional Autonomy. *Sociological Quarterly*, *59*(3), 519–544. <https://doi.org/10.1080/00380253.2018.1479203>
3. Agafonova, R. (2019). International Skating Union versus European Commission: Is the European sports model under threat? *International Sports Law Journal*, *19*(1), 87–101. <https://doi.org/10.1007/s40318-019-00155-6>
4. Allison, L., & Monnington, T. (2002). Sport, prestige and international relations. *Government and Opposition*, *37*(1), 106–134.
5. Alm, J. (2013). Accountability and good governance. *Action for Good Governance in International Sports Organisations*. <https://www.sport.ee/et/file/d0ad60723aeaad8b176d37aa0c364695/taani_2013_final_report_aggis_full_version.pdf#page=22>
6. Baddeley, M. (2020). The extraordinary autonomy of sports bodies under Swiss law: Lessons to be drawn. *International Sports Law Journal*, *20*(1), 3–17. <https://doi.org/10.1007/s40318-019-00163-6>
7. Breuer, C., & Nowy, T. (2017). Germany: Autonomy, partnership and subsidiarity. *Sport Policy Systems and Sport Federations: A Cross-National Perspective*, 157–178.
8. Bruyninckx, H. (2012). Sports governance: Between the obsession with rules and regulation and the aversion to being ruled and regulated. *Sports Governance, Development and Corporate Responsibility*, 107–121.
9. Budevici-puiu, L., & Manolachi, V. (2022). The Autonomy and Specifity of Sport in a National and European Context. *REVISTA ROMANEASCA PENTRU EDUCATIE MULTIDIMENSIONALA*, *14*(3), 457–465. <https://doi.org/10.18662/rrem/14.3/619>
10. Budevici-Puiu, L., Manolachi, V., & Manolachi, V. (2020). Specific Elements of Good Governance in Sport, as Important Factors in Ensuring the Management. *REVISTA ROMANEASCA PENTRU EDUCATIE MULTIDIMENSIONALA*, *12*(4), 328–337. <https://doi.org/10.18662/rrem/12.4/348>
11. Caiger, A., & Gardiner, S. (2000). Professional Sport in the EU: Regulation and Re-regulation. *T.M.C. Asser Press The Hague*. <https://link.springer.com/book/9789067041263>
12. Calo, Z., Moloney, K., & Swart, K. (2023). Legal-administrative implications of international sport for public administration. *Administrative Theory and Praxis*. <https://doi.org/10.1080/10841806.2023.2282923>
13. Carlsson, B., & Lindfelt, M. (2010). Legal and moral pluralism: Normative tensions in a Nordic sports model in transition. *Sport in Society*, *13*(4), 718–733. <https://doi.org/10.1080/17430431003616548>
14. Chappelet, J.-L. (2016). Autonomy and governance: Necessary bedfellows in the fight against corruption in sport. *Global Corruption Report: Sport*, 16–28.
15. Chappelet, J.-L. (2018a). Beyond governance: The need to improve the regulation of international sport. *Sport in Society*, *21*(5), 724–734. <https://doi.org/10.1080/17430437.2018.1401355>
16. Chappelet, J.-L. (2018b). The autonomy of sport and the EU. *Research Handbook on EU Sports Law and Policy*, 157–172.
17. Chappelet, J.-L., & Mrkonjic, M. (2019). Assessing sport governance principles and indicators. *Research Handbook on Sport Governance*, 10–28.
18. Coleman, D. L. (2020). Symposium on the Olympics and international law the Olympic movement in international law. *AJIL Unbound*, *114*, 385–390. <https://doi.org/10.1017/aju.2020.75>
19. Di Marco, A. (2019). The internal governance of sporting organisations: International convergences on an idea of democracy. *Journal Article*, *19*(3), 171–183. <https://doi.org/10.1007/s40318-019-00144-9>
20. Di Marco, A. (2021a). Athletes’ freedom of expression: The relative political neutrality of sport. *Human Rights Law Review*, *21*(3), 620–640. <https://doi.org/10.1093/hrlr/ngab009>
21. Di Marco, A. (2021b). Sports Economy and Fight against Corruption: Which Limits to the Sporting Organisations Autonomy? *European Business Law Review*, *32*(5), 877–904.
22. Di Marco, A. (2022). Human rights in the Olympic Movement: The application of international and European standards to the lex sportiva. *Netherlands Quarterly of Human Rights*, *40*(3), 244–268. <https://doi.org/10.1177/09240519221112554>
23. Dolbysheva, N. (2022). Historical Features of the Development of the Autonomy of non-Olympic Sports in the System of the International Sports Movement in the Period of Modern and Contemporary History. *Sport i Turystyka*, *5*(3), 11–30. <https://doi.org/10.16926/sit.2022.03.01>
24. Donnelly, P., Kerr, G., & Kidd, B. (2022). Contesting the autonomy of sport to realize the right to safe sport: A Canadian case study. *International Sports Law Journal*, *22*(2), 165–170. <https://doi.org/10.1007/s40318-022-00225-2>
25. Dorskaia, A. A., & Dorskii, A. Yu. (2021). Co-regulation as a way to improve the effectiveness of legal regulation in sports. *Vestnik Sankt-Peterburgskogo Universiteta. Pravo*, *12*(2), 263–275. <https://doi.org/10.21638/spbu14.2021.202>
26. Duval, A. (2021). Transnational sports law: The living lex sportiva. *The Oxford Handbook of Transnational Law*, 493–512.
27. Fischer, P., Kornbeck, J., Miège, C., & Stopper, M. (2023). Responsible sport and state oversight: Sports organisations as civil society organisations and private regulators in France and Germany. *International Sports Law Journal*. <https://doi.org/10.1007/s40318-023-00252-7>
28. Flanagan, C. A. (2018). The corridor of uncertainty: Part two, why attempts to regulate the financial aspects of football are met with legal challenges. *International Sports Law Journal*, *18*(1), 29–38. <https://doi.org/10.1007/s40318-018-0125-z>
29. Forster, J. (2006). Global sports organisations and their governance. *Corporate Governance*, *6*(1), 72–83. <https://doi.org/10.1108/14720700610649481>
30. Foster, K. (2004). Alternative models for the regulation of global sport. *The Global Politics of Sport*, 57–78.
31. Foster, K. (2007). The juridification of sport. *Readings in Law and Popular Culture*, 163–190.
32. Foster, K. (2010). Transnational Law in Action. *International Sports Law Review Pandektis*, *3*, 20–25.
33. Foster, K. (2019). Global Sports Law Revisited. *Entertainment & Sports Law Journal*, *17*(2), 1–14. <https://doi.org/10.16997/eslj.228>
34. Foster, K., Caiger, A., & Gardiner, S. (2000). Can Sport be Regulated by Europe?: An Analysis of Alternative Models. *Professional Sport in the EU: Regulation and Re-Regulation*, 43–64.
35. Foster, K., Siekmann, R. C. R., & Soek, J. (2012). Is There a Global Sports Law? *Lex Sportiva: What Is Sports Law?*, 35–52.
36. García, B. (2009). Sport governance after the White Paper: The demise of the European model? *International Journal of Sport Policy and Politics*, *1*(3), 267–284. <https://doi.org/10.1080/19406940903265541>
37. García, B. (2010). The EU and Sport Governance: Between Economic and Social Values. *Social Capital and Sport Governance in Europe*, 21–40.
38. García, B. (2016). From regulation to governance and representation: Agenda-setting and the EU’s involvement in sport. *Entertainment and Sports Law Journal*, *5*(1). <https://doi.org/10.16997/eslj.73>
39. García, B. (2024). Down with the politics, up with the law! Reinforcing EU law’s supervision of sport autonomy in Europe. *International Sports Law Journal*. <https://doi.org/10.1007/s40318-024-00264-x>
40. García, B., & Meier, H. E. (2022). The “autonomy” of developing countries in the Olympic Movement: Assessing the fate of sports governance transplants in the Global South. *Frontiers in Sports and Active Living*, *4*. <https://doi.org/10.3389/fspor.2022.972717>
41. García, B., Meier, H. E., & Moustakas, L. (2023). Racing to Win: Competition and Co-operation between the National Olympic Committee and Public Authorities in the Development of the Botswana Sport System. *Journal of Southern African Studies*, *49*(4), 637–659. <https://doi.org/10.1080/03057070.2023.2289806>
42. García, B., & Weatherill, S. (2012). Engaging with the EU in order to minimize its impact: Sport and the negotiation of the Treaty of Lisbon. *Journal of European Public Policy*, *19*(2), 238–256. <https://doi.org/10.1080/13501763.2011.609710>
43. Geeraert, A. (2014). New EU governance modes in professional sport: Enhancing throughput legitimacy. *Journal of Contemporary European Research*, *10*(3), 302–321.
44. Geeraert, A. (2019). The limits and opportunities of self-regulation: Achieving international sport federations’ compliance with good governance standards. *European Sport Management Quarterly*, *19*(4), 520–538. <https://doi.org/10.1080/16184742.2018.1549577>
45. Geeraert, A., Alm, J., & Groll, M. (2014). Good governance in international sport organizations: An analysis of the 35 Olympic sport governing bodies. *International Journal of Sport Policy & Politics*, *6*(3), 281–306. <https://doi.org/10.1080/19406940.2013.825874>
46. Geeraert, A., Mrkonjic, M., & Chappelet, J.-L. (2015). A rationalist perspective on the autonomy of international sport governing bodies: Towards a pragmatic autonomy in the steering of sports. *International Journal of Sport Policy and Politics*, *7*(4), 473–488. <https://doi.org/10.1080/19406940.2014.925953>
47. Girginov, V. (2019). A cultural perspective on good governance in sport. *Research Handbook on Sport Governance*, 89–101.
48. Girginov, V. (2023). The numbers game: Quantifying good governance in sport. *European Sport Management Quarterly*, *23*(6), 1889–1905. <https://doi.org/10.1080/16184742.2022.2078851>
49. González, C. P. (2022). The effective application of international human rights law standards to the sporting domain: Should UN monitoring bodies take central stage? *International Sports Law Journal*, *22*(2), 152–164. <https://doi.org/10.1007/s40318-021-00209-8>
50. Greitens, M. P. (2021). State and European Law. *Management for Professionals*, 23–41.
51. Halleux, V. (2015). *EU sport policy: An overview*. <https://policycommons.net/artifacts/1336010/eu-sport-policy/1942855/>
52. Harris, S., Dowling, M., & Houlihan, B. (2021). An analysis of governance failure and power dynamics in international sport: The Russian doping scandal. *International Journal of Sport Policy & Politics*, *13*(3), 359–378. <https://doi.org/10.1080/19406940.2021.1898443>
53. Hessert, B. (2021). The protection of minor athletes in sports investigation proceedings. *International Sports Law Journal*, *21*(1), 62–73. <https://doi.org/10.1007/s40318-020-00177-5>
54. Hoye, R. (2013). Sport governance. *Routledge Handbook of Sport Policy*, 331–340.
55. Hylton, J. G. (2017). How FIFA used the principle of autonomy of sport to shield corruption in the Sepp Blatter Era. *Md. J. Int’l L.*, *32*, 134.
56. Ioannidis, G. (2019). Football intermediaries and self-regulation: The need for greater transparency through disciplinary law, sanctioning and qualifying criteria. *International Sports Law Journal*, *19*(3), 154–170. <https://doi.org/10.1007/s40318-019-00159-2>
57. Jedlicka, S. R. (2018). Sport governance as global governance: Theoretical perspectives on sport in the international system. *International Journal of Sport Policy and Politics*, *10*(2), 287–304.
58. Jevtić, B. (2019). Sports system in the independent state of the Republic of Serbia – from the prudent beginning to the exhausting state interventionism. *Physical Culture.*, *73*(1), 3–21. <https://doi.org/10.5937/fizkul1901003J>
59. Johnson, A. T. (1982). Government, opposition and sport: The role of domestic sports policy in generating political support. *Journal of Sport & Social Issues*, *6*(2), 22–34. <https://doi.org/10.1177/019372358200600203>
60. Kalashyan, J. (2022). The game behind the game: UEFA’s Financial Fair Play Regulations and the need to field a substitute. *European Competition Journal*, *18*(1), 21–81. <https://doi.org/10.1080/17441056.2021.1935570>
61. Kornbeck, J. (2022). EU antitrust law and sport governance: The next frontier? *EU Antitrust Law and Sport Governance: The Next Frontier?*, 1.
62. Kruessmann, T. (2019). Extending integrity to third parties: In search of a new model for anti-corruption in sports. *International Sports Law Journal*, *18*(3), 136–149. <https://doi.org/10.1007/s40318-018-0137-8>
63. Lenskyj, H. J. (2018). Gender, Athletes’ Rights, and the Court of Arbitration for Sport. *Emarald Publishing*, 1.
64. Lewandowski, W. (2020). The implications of the recent jurisprudence of the court of justice of the european union for the protection of the fundamental rights of athletes and the regulatory autonomy of sporting federations. *Tilburg Law Review*, *25*(1), 55–66. <https://doi.org/10.5334/TILR.193>
65. Lewis, A., & Taylor, J. (2021). *Sport: Law and practice*. Bloomsbury Publishing. <https://books.google.com/books?hl=en&lr=&id=qG3XEAAAQBAJ&oi=fnd&pg=PR5&dq=(autonomy+OR+self-regulation+OR+self-review+OR+self-governance+OR+freedom+OR+independence+OR+steering)+AND+(sport*+OR+%22sport*+organi*%22+OR+%22sport*+governing+bod*%22+OR+%22sport*+federation*%22+OR+%22sport*+association*%22)&ots=5_LwOLcPv9&sig=piutd6MWbf5NocS0G6P3jfMIOto>
66. Li, M., Hofacre, S., & Mahony, D. (2001). Regulation of sport. *Economics of Sport*. <https://kuleuven.e-bronnen.be/login?url=https://search.ebscohost.com/login.aspx?direct=true&db=sph&AN=SPHS-952819&site=ehost-live&scope=site>
67. McCutcheon, J. P. (2002). Free movement in European sport. *European Sport Management Quarterly*, *2*(4), 308–320.
68. Meeuwsen, S., & Kreft, L. (2023). Sport and Politics in the Twenty-First Century. *Sport, Ethics & Philosophy*, *17*(3), 342–355. <https://doi.org/10.1080/17511321.2022.2152480>
69. Meier, H. E., & García, B. (2021). Beyond sports autonomy: A case for collaborative sport governance approaches. *International Journal of Sport Policy & Politics*, *13*(3), 501–516. <https://doi.org/10.1080/19406940.2021.1905035>
70. Minikin, B. (2015). Legitimacy and democracy: Implications for governance in sport. *Sport, Business and Management: An International Journal*, *5*(5), 435–450. <https://doi.org/10.1108/SBM-03-2015-0010>
71. Modi, T. (2023). To what extent is rule 50 of the Olympic charter valid? Balancing athletes freedom of expression and the mythical political neutrality of sport. *International Sports Law Journal*, *23*(3), 368–389. <https://doi.org/10.1007/s40318-023-00249-2>
72. Mravec, L. (2021). Match-fixing as a Threat to Sport: Ethical and Legal Perspectives. *Studia Sportiva*, *15*(2), 37–48. <https://doi.org/10.5817/STS2021-2-4>
73. Næss, H. E. (2020). The Normative Legitimacy Gap: International Sports Associations, Human Rights and Stakeholder Democracy. *Sport, Ethics & Philosophy*, *14*(2), 129–145. <https://doi.org/10.1080/17511321.2019.1566272>
74. Næss, H. E. (2022). The Neutrality Paradox in Sport: Governance, Politics and Human Rights after Ukraine. *The Neutrality Paradox in Sport: Governance, Politics and Human Rights after Ukraine*, 1.
75. Panagiotopoulos, D. P., & Kallimani, Z. (2017). Appointment of Temporary Administration in the Hellenic Football Federation Greek Government—FIFA Intervention. *Rassegna Di Diritto Ed Economia Dello Sport*, *12*(2), 543–549.
76. Panagiotopoulos, D. P., Mournianakis, J., Alexandrakis, V., & Manarakis, S. (2010). Prospects for EU action in the field of sport after the Lisbon treaty. *International Sports Law Review Pandektis*, *8*(3), 301–310.
77. Papaloukas, M. (2013). Sports Self-Governance. *International Sports Law Review Pandektis*, *10*(1), 95–104.
78. Parrish, R. (2002). Judicial intervention and sporting autonomy: Defining the territories of European union involvement in sport. *European Sport Management Quarterly*, *2*(4), 296–307.
79. Parrish, R. (2012). Lex sportiva and EU sports law. *European Law Review*, *37*(6), 716–733.
80. Parrish, R., & McArdle, D. (2004). Beyond Bosman: The European Union’s Influence upon Professional Athletes’ Freedom of Movement. *Sport in Society*, *7*(3), 403–419. <https://doi.org/10.1080/1743043042000291712>
81. Pearson, G. (2015). Sporting justifications under EU free movement and competition law: The case of the football ‘Transfer System’. *European Law Journal*, *21*(2), 220–238. <https://doi.org/10.1111/eulj.12110>
82. Pijetlovic, K. (2010). Another classic of EU sports jurisprudence: Legal implications of olympique lyonnais sasp v olivier bernard and newcastle UFC (C-325/08). *European Law Review*, *35*(6), 857–868.
83. Ponkina, A. I. (2013). Autonomy of sport: Legal aspects. *International Sports Law Review Pandektis*, *10*(1), 204–215.
84. Porat, A. B. (2019). Football ‘made in Israel’. *Israel Studies Review*, *34*(3), 1–16. <https://doi.org/10.3167/isr.2019.340302>
85. Rook, W., Prado, T., & Heerdt, D. (2023). Responsible sport: No going back. *International Sports Law Journal*, *23*(1), 85–98. <https://doi.org/10.1007/s40318-022-00231-4>
86. Sárközy, T. (2001). Regulation in sport as a borderline case between state and law regulation and self-regulation. *Acta Juridica Hungarica*, *42*(3), 159–180.
87. Scelles, N., Scheerder, J., Willem, A., & Claes, E. (2017). France: Organisation of Sport and Policy Towards Sport Federations. *Sport Policy Systems and Sport Federations*, 135–155.
88. Scheerder, J. (2020). Conclusion: Established models of European sport revisited from a socio-politological approach1. *Sport, Welfare and Social Policy in the European Union*, 153–168.
89. Scheerder, J., Claes, E., Willem, A., Scheerder, J., Willem, A., & Claes, E. (2017). Does It Take Two to Tango? The Position and Power of National Sport bodies Compared to Their Public Authorities. *Sport Policy Systems and Sport Federations*, 1–17.
90. Schimank, U. (2005). The autonomy of modern sport: Dangerous and endangered. *European Journal for Sport and Society*, *2*(1), 13–23. <https://doi.org/10.1080/16138171.2005.11687762>
91. Schwab, B. (2018). ‘Celebrate Humanity’: Reconciling Sport and Human Rights Through Athlete Activism. *Journal of Legal Aspects of Sport*, *28*(2), 170–207. <https://doi.org/10.18060/22570>
92. Serby, T. (2015). The council of europe convention on manipulation of sports competitions: The best bet for the global fight against match-fixing? *International Sports Law Journal*, *15*(1), 83–100. <https://doi.org/10.1007/s40318-015-0069-5>
93. Serby, T. (2016). The state of EU sports law: Lessons from UEFA’s ‘Financial Fair Play’ regulations. *International Sports Law Journal*, *16*(1), 37–51. <https://doi.org/10.1007/s40318-016-0091-2>
94. Serby, T. (2017). Sports Corruption: Sporting Autonomy, Lex Sportiva and the Rule of Law. *Entertainment & Sports Law Journal*, *15*(1), 1–9. <https://doi.org/10.16997/eslj.204>
95. Serra, M. F. (2020). Lex sportiva: Present and future perspective. *International Sports Law Review Pandektis*, *13*(1), 126–135.
96. Shevchenko, O. A., Ponkin, I. V., & Ponkina, A. I. (2016). Limits of intervention and immunities of international sport: Case study of FIFA issue in 2015. *International Sports Law Review Pandektis*, *11*(3), 336–344.
97. Shinohara, T. (2022). Which states parties should be held responsible for the implementation of positive obligations under the ECHR in sports-related disputes? *International Sports Law Journal*, *22*(4), 332–342. <https://doi.org/10.1007/s40318-021-00202-1>
98. Szatkowski, M. (2022). Analysis of the sports model in selected Western European countries. *Journal of Physical Education & Sport*, *22*(3), 829–839. <https://doi.org/10.7752/jpes.2022.03105>
99. Szwedo, P. (2011). Poland at the Gates of EURO 2012—Global Sport Governance and the Limits of the State’s Autonomy. *University of Denver Sports & Entertainment Law Journal*, 57–80.
100. Taylor, T. (1988). Sport and World Politics: Functionalism and the State System. *International Journal*, *43*(4), 531–553.
101. Thing, L. F., & Ottesen, L. (2010). The autonomy of sports: Negotiating boundaries between sports governance and government policy in the Danish welfare state. *International Journal of Sport Policy and Politics*, *2*(2), 223–235. <https://doi.org/10.1080/19406940.2010.488070>
102. Thompson, A., Lachance, E. L., Parent, M. M., & Hoye, R. (2023). A systematic review of governance principles in sport. *European Sport Management Quarterly*, *23*(6), 1863–1888. <https://doi.org/10.1080/16184742.2022.2077795>
103. van der Walt, J. C. (1982). Autonomy in sport and South African statutory law: A critical evaluation. *South African Journal for Research in Sport, Physical Education & Recreation (SAJR SPER)*, *5*(1), 91=103-91=103.
104. Vieweg, K. (2000). The legal autonomy of sport organisations and the restrictions of European law. *Professional Sport in the EU: Regulation and Re-Regulation*, 83–106.
105. Vieweg, K. (2014). Lex sportiva and the fairness principle. *International Sports Law Review Pandektis*, *10*(3), 382–394.
106. Waters, L. (2023). In Pursuit of Prestige: The International Olympic Committee’s Peace Efforts in Bosnia-Herzegovina (1992–1994). *International Journal of the History of Sport*, *40*(15), 1347–1363. <https://doi.org/10.1080/09523367.2024.2312153>
107. Weatherill, S. (2012). EU Sports Law: The Effect of the Lisbon Treaty. *EU Law after Lisbon*. <https://www.scopus.com/inward/record.uri?eid=2-s2.0-84920505984&doi=10.1093%2facprof%3aoso%2f9780199644322.003.0019&partnerID=40&md5=529a6e2ec99736c336223f38780ef1db>
108. Weatherill, S. (2017). *Principles and Practice in EU Sports Law*. Oxford University Press.
109. Weatherill, S. (2022). Saving Football from Itself: Why and How to Re-make EU Sports Law. *Cambridge Yearbook of European Legal Studies*, *24*, 4–23. <https://doi.org/10.1017/cel.2022.3>
110. Wiater, P. (2023). Chaos in the Sporting World over Russia’s War of Aggression: Political Neutrality in Light of Human Rights Protection. *Business and Human Rights Journal*, *8*(3), 461–467. <https://doi.org/10.1017/bhj.2023.32>
111. Winand, M., Steen, A., & Kasale, L. L. (2023). Performance Management Practices in the Sport Sector: An Examination of 32 Scottish National Sport Organizations. *Journal of Global Sport Management*, *8*(4), 739–762. <https://doi.org/10.1080/24704067.2021.1899765>
112. Xiang, H. (2017). CAS Jurisprudence on Match-fixing under the Context of Domestic Criminal Law Intervention. *Rassegna Di Diritto Ed Economia Dello Sport*, *12*(2), 429–436.
113. Yaghi, A., & Almutawwa, R. (2023). Perceptions of Sport Governance and Performance in United Arab Emirates. *Public Organization Review*, *23*(1), 113–131. <https://doi.org/10.1007/s11115-022-00631-y>
114. Zakharova, L. I., & Melnik, T. E. (2020). Some reflections on regulating professional sports in integration associations: A sphere of autonomy or an object of the union law regulation? *Comparative Law Review*, *26*, 255–279. <https://doi.org/10.12775/CLR.2020.011>
115. Zeimers, G., Lefebvre, A., Winand, M., Anagnostopoulos, C., Zintz, T., & Willem, A. (2021). Organisational factors for corporate social responsibility implementation in sport federations: A qualitative comparative analysis. *European Sport Management Quarterly*, *21*(2), 173–193. <https://doi.org/10.1080/16184742.2020.1731838>
116. Zintz, T., & Gérard, S. (2019). Support the implementation of good governance in sport (SIGGS): A European project for national Olympic committees and national sport federations. *Research Handbook on Sport Governance*, 53–71.
117. IOC. (2009). XIII Olympic Congress Copenhagen. <https://stillmed.olympic.org/media/Document%20Library/OlympicOrg/IOC/Congresses/XIII-Olympic-Congress-Copenhagen-2009/Overview/EN-XIII-Olympic-Congress-Proceedings.pdf>

***B. List of included records from web search***

Alvad, S., & Wickstrøm, M. (2017). *Autonomy in National Olympic Committees 2017. An autonomy index.* Play the Game.

ASOIF. (2019). *Future of Global Sport*.

ASOIF. (2022). *The Solidarity Model of Organised Sport in Europe and Beyond—A Stable Platform For Collaboration*.

ASOIF. (2023a). *40 Years of ASOIF*.

ASOIF. (2023b). *Guidance notes for International Federations—Governance Obligations of  National and Continental Member Federations*.

1. Bach, T. (2013). Statement on the occasion of the adoption of the resolution “Building a peaceful and better world through sport and the Olympic ideal”. https://stillmed.olympic.org/Documents/IOC_President/2013- 11-%206_Speech_IOC_President_Bach-%20Olympic_Truce_adoption_Speech_4_November.pdf [Accessed September 9, 2024].

Chappelet, J.-L. (2010). *Autonomy of sport in Europe*. Council of Europe.

Choi, S. (2023). *Enhancement Plan to Increase Self Revenue  for the Korean Sport & Olympic Committee, securing and strengthening Autonomy—Focusing on analysis of NOC’s financial statement*.

Colucci, M., & Geeraert, A. (2013). *The ‘Social dialogue’ in European professional football*.

Cornu, P., Cuendet, S., & Vidal, L. (2017). Disciplinary And Arbitration Procedures Of The Sport Movement. *Council Of Europe*.

1. Council of Europe. (1992). *Revised European Sports Charter*. Strasbourg: Council of Europe. https://edoc.coe.int/en/sport-for-all/11299-revised-european-sports-charter.html?utm_source=chatgpt.com [Accessed October 18, 2024].

Council of Europe. (2005). *Recommendation of the Committee of Ministers to member states on the principles of good governance in sport (Adopted by the Committee of Ministers on 20 April 2005 at the 924th meeting of the Ministers’ Deputies)*. <https://search.coe.int/cm?i=09000016805b017f>

Council of Europe. (2008). *Resolution: The need to preserve the European Sport Model (1602 (2008))*. <https://assembly.coe.int/nw/xml/XRef/Xref-XML2HTML-en.asp?fileid=17628&lang=en>

Council of Europe. (2011). *Recommendation of the Committee of Ministers to member states on the principle of autonomy of sport in Europe (Adopted by the Committee of Ministers on 2 February 2011 at the 1104th meeting of the Ministers’ Deputies)*. <https://search.coe.int/cm?i=09000016805b4d00>

1. Council of Europe. (2014). *The Convention on the Manipulation of Sport Competitions (the Macolin Convention)*. Available at: <https://rm.coe.int/16801cdd7esport> [Accessed February 24, 2025].

Court of Arbitration for Sport. (2005). CAS 2005/A/1001 Fulham FC (1987) Ltd v. Fédération Internationale de Football Association (FIFA), award of 9 May 2006. https://jurisprudence.tas-cas.org/Shared%20Documents/1001.pdf

Court of Arbitration for Sport. (2005). *CAS 2005/A/847 Hans Knauss v. FIS, award of 20 July 2005.*<https://jurisprudence.tas-cas.org/Shared%20Documents/847.pdf> [Accessed February 28, 2025].

Court of Arbitration for Sport. (2011). *CAS 2011/O/2422 United States Olympic Committee (USOC) v. International Olympic Committee (IOC), award of 4 October 2011.* [*https://jurisprudence.tas-cas.org/Shared%20Documents/2422.pdf*](https://jurisprudence.tas-cas.org/Shared%20Documents/2422.pdf)

Court of Arbitration for Sport. (2012). *CAS 2011/A/2425 Ahongalu Fusimalohi v. Fédération Internationale de Football Association (FIFA), award of 8 March 2012.* [*https://jurisprudence.tas-cas.org/Shared%20Documents/2425.pdf*](https://jurisprudence.tas-cas.org/Shared%20Documents/2425.pdf) [Accessed February 28, 2025].

Court of Arbitration for Sport. (2012). *CAS 2011/A/2426 Amos Adamu v. Fédération Internationale de Football Association (FIFA), award of 24 February 2012. https://jurisprudence.tas-cas.org/Shared%20Documents/2426.pdf*

Court of Arbitration for Sport. (2012). *CAS 2011/A/2658 British Olympic Association (BOA) v. World Anti-Doping Agency (WADA), award of 30 April 2012. https://jurisprudence.tas-cas.org/Shared%20Documents/2658.pdf*

Court of Arbitration for Sport. (2013). *CAS 2013/A/3298 Chippa United FC v. South African Football Association (SAFA), The National Soccer League (NSL), Mpumalanga Black Aces FC, Santos FC, Polokwane City FC, Thanda Royal Zulu FC (Pty) Limited, Adv. P. Pretorius SC N.O., award of 27 June 2014. https://jurisprudence.tas-cas.org/Shared%20Documents/3298.pdf*

Court of Arbitration for Sport. (2015). *CAS 2014/A/3828 Indian Hockey Federation (IHF) v. International Hockey Federation (FIH) & Hockey India, award of 17 September 2015. https://jurisprudence.tas-cas.org/Shared%20Documents/3828.pdf*

Court of Arbitration for Sport. (2015). *CAS 2015/A/3926 FC Gelsenkirchen-Schalke 04 v. Union des Association Européennes de Football (UEFA), award of 6 October 2015. https://jurisprudence.tas-cas.org/Shared%20Documents/3926.pdf*

Court of Arbitration for Sport. (2016). CAS 2014/A/3776 Gibraltar Football Association (GFA) v. Fédération Internationale de Football Association (FIFA), award of 27 April 2016. https://jurisprudence.tas-cas.org/shared%20documents/3776.pdf

Court of Arbitration for Sport. (2016). *CAS 2016/A/4701 Weightlifting Federation of the Republic of Kazakhstan (WFRK) v. International Weightlifting Federation (IWF), award of 10 March 2017. https://jurisprudence.tas-cas.org/Shared%20Documents/4701.pdf*

Court of Arbitration for Sport. (2016). *CAS 2016/A/4722 ACS Poli Timisoara v. Romanian Football Federation (RFF) & Romanian Professional Football League (RPFL), award of 2 March 2017. https://jurisprudence.tas-cas.org/Shared%20Documents/4722.pdf*

Court of Arbitration for Sport. (2016). *CAS 2016/A/4812 Bulgarian Chess Federation v. European Chess Union (ECU), award of 22 May 2017. https://jurisprudence.tas-cas.org/Shared%20Documents/4812.pdf*

Court of Arbitration for Sport. (2017). *CAS 2016/A/4698 Belarusian Weightlifting Union (BWU) v. International Weightlifting Federation (IWF), award dated 10 March 2017.* [*https://jurisprudence.tas-cas.org/Shared%20Documents/4698.pdf*](https://jurisprudence.tas-cas.org/Shared%20Documents/4698.pdf)

Court of Arbitration for Sport. (2017). *CAS 2017/A/4947 Ion Viorel v. Romanian Football Federation (RFF), award of 6 October 2017.* [*https://jurisprudence.tas-cas.org/Shared%20Documents/4947.pdf*](https://jurisprudence.tas-cas.org/Shared%20Documents/4947.pdf)

Court of Arbitration for Sport. (2017). *CAS 2017/A/5117 Eskisehir Spor Kulübü v. Ibrahim Sissoko & Fédération Internationale de Football Association (FIFA), award of 1 March 2018.* [*https://jurisprudence.tas-cas.org/Shared%20Documents/5117.pdf*](https://jurisprudence.tas-cas.org/Shared%20Documents/5117.pdf)

Court of Arbitration for Sport. (2017). *CAS 2017/A/5117 Eskisehir Spor Kulübü v. Ibrahim Sissoko & Fédération Internationale de Football Association (FIFA), award of 1 March 2018. https://jurisprudence.tas-cas.org/Shared%20Documents/5117.pdf*

Court of Arbitration for Sport. (2018). *CAS 2017/A/5003 Jérôme Valcke v. FIFA. <https://www.tas-cas.org/fileadmin/user_upload/Award_5003_Final.pdf>*

Court of Arbitration for Sport. (2018). *CAS 2018/A/5622 Londrina Esporte Clube v. Fédération Internationale de Football Association (FIFA), award of 7 August 2018.* [*https://jurisprudence.tas-cas.org/Shared%20Documents/5622.pdf*](https://jurisprudence.tas-cas.org/Shared%20Documents/5622.pdf)

Court of Arbitration for Sport. (2018). *CAS 2018/A/5888 Centro Atlético Fénix, Club Atlético Boston River, Club Atlético Cerro, Club Atlético Progreso, Club Atlético River Plate, Danubio Fútbol Club, Defensor Sporting Club, Liverpool Fútbol Club, Cerro Largo FC, Central Español Fútbol Club, Club Atlético Villa Teresa, Racing Club de Montevideo, Club Sportivo Miramar Misiones, Montevideo Wanderers F.C., Club Atlético Juventud v. Fédération Internationale de Football Association (FIFA) & Confederación Sudamericana de Fútbol (CONMEBOL) & Asociación Uruguaya de Fútbol (AUF), award of 25 June 2019.* [*https://jurisprudence.tas-cas.org/Shared%20Documents/5888.pdf*](https://jurisprudence.tas-cas.org/Shared%20Documents/5888.pdf)

Court of Arbitration for Sport. (2018). *CAS 2018/O/5830 International Surfing Association (ISA) v. International Canoe Federation (ICF), award of 5 August 2020. https://jurisprudence.tas-cas.org/Shared%20Documents/5830.pdf*

Court of Arbitration for Sport. (2019). *CAS 2019/A/6278 Cruzeiro EC v. Fédération Internationale de Football Association (FIFA), award of 16 December 2019. https://jurisprudence.tas-cas.org/Shared%20Documents/6278.pdf*

Court of Arbitration for Sport. (2019). *CAS 2019/A/6330 Sara Castillo Martínez v. World Skate, award of 18 February 2020 (operative part of 9 July 2019). https://jurisprudence.tas-cas.org/Shared%20Documents/6330.pdf*

Court of Arbitration for Sport. (2020). *CAS 2017/O/5264, 5265 & 5266 Miami FC & Kingston Stockade FC v. Fédération Internationale de Football Association (FIFA), Confederation of North, Central America and Caribbean Association Football (CONCACAF) & United States Soccer Federation (USSF), award of 3 February 2020.* [*https://jurisprudence.tas-cas.org/Shared%20Documents/5264,%205265,%205266.pdf*](https://jurisprudence.tas-cas.org/Shared%20Documents/5264,%205265,%205266.pdf)

Court of Arbitration for Sport. (2020). *CAS 2017/O/5264*. CAS 2017/O/5264, 5265 & 5266 Miami FC & Kingston Stockade FC v. Fédération Internationale de Football Association (FIFA), Confederation of North, Central America and Caribbean Association Football (CONCACAF) & United States Soccer Federation (USSF), award of 3 February 2020. <https://jurisprudence.tas-cas.org/Shared%20Documents/5264,%205265,%205266.pdf>

Court of Arbitration for Sport. (2020). *CAS 2018/A/6072 Kwesi Nyantakyi v. Fédération Internationale de Football Association (FIFA), award of 9 April 2020.* [*https://jurisprudence.tas-cas.org/Shared%20Documents/6072.pdf*](https://jurisprudence.tas-cas.org/Shared%20Documents/6072.pdf)

Court of Arbitration for Sport. (2020). *CAS 2020/A/7090 Club Universidad de Guadalajara, Venados FC Yucatán & CF Correcaminos v. Federación Mexicana de Fútbol & Mexican Liga MX/Liga Ascenso MX. https://www.tas-cas.org/fileadmin/user_upload/Award_Final_7090_for_publication.pdf*

Court of Arbitration for Sport. (2020). *CAS 2020/A/7346 Neimenggu Zhongyou Football Club v. FIFA. https://www.tas-cas.org/fileadmin/user_upload/Bulletin_TAS_2023-1_4.pdf*

Court of Arbitration for Sport. (2021). *CAS 2019/A/6665 Ricardo Terra Teixeira v. Fédération Internationale de Football Association. https://www.tas-cas.org/fileadmin/user_upload/CAS_Award_6665__FINAL__for_publication.pdf*

Court of Arbitration for Sport. (2021). *CAS 2020/A/7549 Singapore Karate-do Federation (SKF) v. World Karate Federation (WKF), award of 9 November 2021. https://jurisprudence.tas-cas.org/Shared%20Documents/7549.pdf*

Court of Arbitration for Sport. (2022). *CAS 2022/A/8708 Football Union of Russia v. Fédération Internationale de Football Association et al. https://www.tas-cas.org/fileadmin/user_upload/8708_FINAL_Award__FINAL_.pdf*

Court of Arbitration for Sport. (2022). *CAS 2022/A/8709 Football Union of Russia (FUR) v. Union of European Football Associations et al. https://www.tas-cas.org/fileadmin/user_upload/8709_reasoned_OPM__publication_.pdf*

Court of Arbitration for Sport. (2022). *CAS 2022/A/8731 Shanghai Shenhua FC v. Fédération Internationale de Football Association (FIFA), award of 28 March 2023. https://jurisprudence.tas-cas.org/Shared%20Documents/8731.pdf*

Court of Arbitration for Sport. (2022). *CAS 2022/A/8865 FC Zenit JSC v. Union des Associations Européennes de Football (UEFA). https://www.tas-cas.org/fileadmin/user_upload/Award_8865_-_8868__FINAL_.pdf*

Court of Arbitration for Sport. (2022). *CAS 2022/A/8871 Football Union of Russia (FUR) v. Union of European Football Associations (UEFA) et al., award of 25 November 2022. https://editorial.uefa.com/resources/028b-1a64869a1c31-358efc1abe50-1000/cas_2022a8871_football_union_of_russia_fur_v._uefa.pdf*

Court of Arbitration for Sport. (2022). *CAS 2022/A/9282 Al Batin Club v. Mohamed Rayhi & FIFA. https://www.tas-cas.org/fileadmin/user_upload/CAS_9282_Award.pdf*

Court of Arbitration for Sport. (2023). *CAS 2022/A/9016 FC Shakhtar Donetsk v. Fédération Internationale de Football Association (FIFA), award of 3 May 2023 (operative part of 13 January 2023). https://jurisprudence.tas-cas.org/Shared%20Documents/9016.pdf*

Court of Arbitration for Sport. (2023). *CAS 2022/A/9016 FC Shakhtar Donetsk v. Fédération Internationale de Football Association (FIFA), award of 3 May 2023 (operative part of 13 January 2023).* [*https://jurisprudence.tas-cas.org/Shared%20Documents/9016.pdf*](https://jurisprudence.tas-cas.org/Shared%20Documents/9016.pdf)

1. Court of Justice of the European Union. (1974). *BNO Walrave and LJN Koch v Association Union Cycliste Internationale, Koninklijke Nederlandsche Wielren Unie and Federación Española Ciclismo*, Case 36-74. EUR-Lex. <https://eur-lex.europa.eu/legal-content/EN/TXT/?uri=CELEX%3A61974CJ0036> [Accessed February 28, 2025].
2. Court of Justice of the European Union. (1995). *Union Royale Belge Des Sociétés de Football Association ASBL v Jean-Marc Bosman, Royal Club Liégeois SA v Jean-Marc Bosman and Others and Union Des Associations Européennes de Football (UEFA) v Jean-Marc Bosman*, Case C-415/93. EUR-Lex. <https://eur-lex.europa.eu/legal-content/EN/TXT/?uri=CELEX:61993CJ0415> [Accessed February 28, 2025].
3. Court of Justice of the European Union. (2006). *David Meca-Medina and Igor Majcen v Commission of the European Communities*, Case C-519/04 P. EUR-Lex. <https://eur-lex.europa.eu/legal-content/EN/TXT/?uri=CELEX%3A62004CJ0519> [Accessed February 28, 2025].
4. Court of Justice of the European Union. (2008). *Motosykletistiki Omospondia Ellados NPID (MOTOE) v Elliniko Dimosio*, Case C-49/07. EUR-Lex. <https://eur-lex.europa.eu/legal-content/en/TXT/?uri=CELEX:62007CJ0049> [Accessed February 28, 2025].
5. Court of Justice of the European Union. (2010). *Olympique Lyonnais SASP v Olivier Bernard and Newcastle UFC*, Case C-325/08. EUR-Lex. <https://eur-lex.europa.eu/legal-content/en/TXT/?uri=CELEX:62008CJ0325> [Accessed February 28, 2025].
6. Court of Justice of the European Union. (2023). *European Superleague Company, SL v Fédération Internationale de Football Association (FIFA) and Union of European Football Associations (UEFA)*, Case C-333/21. EUR-Lex. <https://eur-lex.europa.eu/legal-content/en/TXT/?uri=CELEX:62021CJ0333> [Accessed February 28, 2025].
7. Court of Justice of the European Union. (2023). *International Skating Union v European Commission*, Case C-124/21 P. EUR-Lex. <https://eur-lex.europa.eu/legal-content/EN/TXT/?uri=CELEX%3A62021CJ0124> [Accessed February 28, 2025].
8. Court of Justice of the European Union. (2023). *UL and SA Royal Antwerp Football Club v Union Royale Belge Des Sociétés de Football Association ASBL*, Case C-680/21. EUR-Lex. <https://eur-lex.europa.eu/legal-content/EN/TXT/?uri=CELEX%3A62021CJ0680> [Accessed February 28, 2025].
9. Court of Justice of the European Union. (2024). *Fédération Internationale de Football Association (FIFA) v BZ*, Case C-650/22. EUR-Lex. <https://eur-lex.europa.eu/legal-content/en/TXT/?uri=CELEX:62022CJ0650> [Accessed February 28, 2025].

European Parliament. (2008). *Resolution of 8 May 2008 on the White Paper on Sport* ([2007/2261(INI)](https://oeil.secure.europarl.europa.eu/oeil/popups/ficheprocedure.do?lang=en&reference=2007/2261(INI))). <https://www.europarl.europa.eu/doceo/document/TA-6-2008-0198_EN.html> [Accessed February 24, 2025].

European Parliament. (2015). *Resolution of 11 June 2015 on recent revelations on high-level corruption cases in FIFA* (2015/2730(RSP)). <https://eur-lex.europa.eu/legal-content/EN/TXT/?uri=CELEX%3A52015IP0233> [Accessed February 24, 2025].

European Parliament. (2017). *Resolution of 2 February 2017 on an integrated approach to Sport Policy: good governance, accessibility and integrity* (2016/2143(INI)). <https://eur-lex.europa.eu/legal-content/EN/TXT/?uri=CELEX%3A52017IP0012> [Accessed February 24, 2025].

FIFA. (2023). *FIFA Commentary on the FIFA Regulations for the Status and Transfer of Players*.

FIFA. (2024). *FIFA Legal Handbook*.

Geeraert, A. (2018). *National Sports Governance Observer. Final report.* Play the Game.

1. IOC. (1949). *Olympic Rules*.

IOC. (2008). *Basic Universal Principles of Good Governance of the Olympic and Sports Movement. Seminar on Autonomy of Olympic and Sport Movement, 11- 12 February 2008*.

IOC. (2021). *Olympic Agenda 2020. Closing report.*

IOC. (2022). *Basic Universal Principles  of Good Governance within  the Olympic Movement*.

IOC. (2024a). *IOC Code of Ethics*.

IOC. (2024b). *IOC Olympic Charter*.

Jack, R. (2019). *Good Governance Initiatives In Sport At National Level*.

Mrkonjic, M. (2013). *AGGIS: The Swiss Regulatory Framework and International Sports Organisations*.

SIGGS. (2019). *Roadmap Principle 2: Autonomy & Accountability*.

UEFA. (2019). *Together for the Future of Footbal. UEFA Strategy 2019-2024.*

1. UN. (2003). *Convention Against Transnational Organized Crime*. Available at: <https://www.unodc.org/unodc/en/organized-crime/intro/UNTOC.html> [Accessed February 24, 2025].
2. UN. (2011). *Guiding Principles on Business and Human Rights: Implementing the United Nations "Protect, Respect and Remedy" Framework*. Available at: https://www.ohchr.org/en/issues/business/pages/businessindex.aspx [Accessed February 24, 2025].
3. UNESCO. (2005). *International Convention against Doping in Sport*. Available at: <https://www.wada-ama.org/en/resources/unesco-international-convention-against-doping-sport> [Accessed February 24, 2025].

UNESCO. (2013). *Declaration Of Berlin Of The Fifth International Conference Of Ministers And Senior Officials Responsible For Physical Education And Sport (MINEPS V)*.

1. UNESCO. (2015). Final Report of the Intergovernmental Committee for Physical Education and Sport, 29–30 January 2015 (CIGEPS/2015/INF.REV) <https://unesdoc.unesco.org/ark:/48223/pf0000232512> [Accessed February 24, 2025].

UNESCO. (2017). *KAZAN ACTION PLAN. The Ministers meeting at the Sixth International Conference of Ministers and  Senior Officials Responsible for Physical Education and Sport (MINEPS VI),  held in Kazan (13-15 July 2017),*.

UNESCO. (2023). *Guidelines on sport integrity*.

UNODC. (2019). *Safeguarding Sport From Corruption. Towards effective Implementation of  resolution 7/8 on corruption in sport.*

UNODC. (2021). *Global Report on Corrupion in Sport*.

Wenn, S. R. (2024). *Financing the Olympic Movement—Early  developments and  evolutions*. The Olympic Studies Centre.
