# Supplementary material for: The autonomy of sport concept: a scoping review
Source: Front Sports Act Living. 2025 Jun 19;7:1593673. doi: 10.3389/fspor.2025.1593673 (PMC12222117; doi:10.3389/fspor.2025.1593673)
Supplement: Supplementary Material 5 — Extraction guidance sheet for a systematic scoping review. [file Table5.docx]

Supplementary Material 5. Extraction guidance sheet for a systematic scoping review.

| *Study characteristics* | |
| --- | --- |
| Author | Eg, Smith; Smith & Hunt; Smith et al. (for more than 2 authors) |
| Title | What is the title of this article, report, etc? Write the full title. |
| Year | The year the record was published |
| Publication type | - Journal article - Book - Book chapter - Conference abstract - Thesis - Editorial - Report (incl. white paper) - Policy (incl. resolution, recommendation) - Statute - Press release - Court Ruling |
| Publication source | Where was this record published (e.g., Routledge, International Sports Law Journal). If it is an organization policy, write the organization (e.g., European Commission, ASOIF). |
| First Author – Country of University Affiliation | What is the country of university affiliation of the first author? |
| Country of study context | - UK - USA - .. - Multicountry - Nonspecified |
| Type of organization (population) | - Multilevel - International multi-sport governing body - National multi-sport governing body (National Olympic Committees) - International sport governing body - National sport governing body - Professional club - Undefined |
| Sport | - Football - Basketball - .. - Multisport - Undefined |
| Research Design | - Non-empirical - Empirical |
| Theoretical Framework^^[[1]](#footnote-1)^^ | - Write theoretical Framework(s) used - N/A |
| Empirical Design | - Qualitative - Quantitative |
| Data collection strategy (if empirical) | - Questionnaires - Interviews - Focus groups - … |
| *Key findings relevant to the research questions* | |
| Definition of autonomy of sport (if provided) | How the record defines the autonomy of sport? |
| Dimentions of autonomy of sport addressed in the study - as defined by Geeraert et al. (2015) political, legal, financial, pyramidal | Which dimention(s) of autonomy discussed in the record? |
| Research Topic | In which research topic the autonomy discussed? (e.g., Bosman, European Super League, sport policy, governance) |
| Stakeholders | Which stakeholders discussed? Write the stakeholders, observe the link to the autonomy between stakeholders (e.g., IOC – IF - NF) |
| Limits/prerequisites to autonomy of sport (if provided) | What are the limits/ prerequisites to autonomy of sport observed in the record? |
| Developments affecting autonomy of sport (if provided) | What are the developments affecting autonomy of sport outlined in the record? |
| Counterstrategies to the threats of autonomy (if provided) | What are the counterstrategies to the threats outlined? |
| Research agenda | What are the future directions and research agenda reported in the record? |

1. Theoretical frameworks refer to a structural representation of relationships between concepts (Doherty, 2013) [↑](#footnote-ref-1)
