# Supplementary material for: The autonomy of sport concept: a scoping review
Source: Front Sports Act Living. 2025 Jun 19;7:1593673. doi: 10.3389/fspor.2025.1593673 (PMC12222117; doi:10.3389/fspor.2025.1593673)
Supplement: Supplementary Material 6 — Summary of Extracted Data. [file Table6.docx]

Supplementary Material 6: Summary of Extracted Data

| **Author, Year (Type of Publication type; First author - Counrty of University Affiliation)** | **Publication Source** | **Research Design** | | **Theoretical Framework*** | **Use of Dimensions Autonomy Political (Pol), Legal (L), Financial (F) and/or Pyramidal (Pyr)** | **Sport type** | **Country of study context** | **Type of sport organisation** |
| --- | --- | --- | --- | --- | --- | --- | --- | --- |
|  |  | **Non- Empirical** | **Empirical** | |  |  |  |  |
| Geeraert et al., 2015 (Journal article; Belgium) | International Journal of Sport Policy and Politics | X |  | No | Pol, L, F, Pyr | Football | Multicountry | ISO |
| Girginov, 2019 (Book chapter; UK) | Edward Elgar Publishing | X |  | Max Weber's theory, new instituonal economics, cultural value dimensions | Pol | Multisport | Multicountry | ISO |
| Thompson et at., 2023  (Journal article;  Canada) | European Sport Management Quarterly |  | Systematic review | No | Pol, L, F, Pyr | Multisport | Multicountry | ISO |
| Alm ed. et al., 2013  (Report; Sweden) | Play the Game/Danish Institute for Sports Studies | X |  | No | Pol, L, F, Pyr | Multisport | Multicountry | ISO |

| Foster, 2004 (Book chapter; UK) | Routledge | X |  | No | Pol, L, F, Pyr | Football | Multicountry | ISO |
| --- | --- | --- | --- | --- | --- | --- | --- | --- |
| Harris et al.,  2021 (Journal article; USA) | International Journal of Sport Policy and Politics |  | Qualitative Documental Analysis | Governance theory, Bergsgard’s analytical framework of power dimensions | Pol, L | Multisport | Russia | ISO |
| Szatkowski et al., 2022  (Journal article;  Poland) | Journal of Physical Education & Sport |  | Formal and dogmatic analysis of legal provisions | No | Pol, L, F, Pyr | Multisport | Multicountry (Germany, Great Britain, France, Italy, Spain) | NSO |
| Pijetlovic, 2019  (Journal article; UK) | European Law Review | X |  | No | L | Football | Multicountry | ISO |
| Chappelet & Mrkonjic, 2019 (Book chapter;  Switzerland) | Edward Elgar Publishing | X |  | No | Pol, L, F, Pyr | Multisport | Multicountry | ISO |
| Di Marco, 2021  (Journal article;  France) | Human Rights Law Review | X |  |  | Pol, L | Multisport | Multicountry | ISO |

| Chappelet, 2016 (Report; Switzerland) | Transparency International | X |  | No | Pol, L | Multisport | Multicountry | ISO, NSO |
| --- | --- | --- | --- | --- | --- | --- | --- | --- |
| van der Walt, 1982 (Journal article;  South Africa) | South African Journal for Research in Sport, Physical Education & Recreation | X |  | No | Pol, L, F | Multisport | South Africa | NSO |
| Ponkina, 2013  (Journal article; Russia) | International Sports Law Review Pandektis | X |  | No | Pol, L, F, Pyr | Multisport | Multicountry | ISO, NSO |
| Parrish & McArdle, 2004 (Journal article;  UK) | Sport in Society | X |  | No | L | Football | EU | ISO |
| Chappelet, 2018 (Journal article;  Switzerland) | Sport in Society | X |  | Pérez’s governance model | Pol, L, F, Pyr | Multisport | Multicountry | ISO |
| Meier & García, 2021  (Journal article;  Germany) | International Journal of Sport Policy and Politics | X |  | Collaborative governance theory | Pol, L, F, Pyr | Multisport | Multicountry | ISO |

| Foster, 2000 (Book chapter; UK) | T.M.C. Asser Press | X |  | No | Pol, L, P | Multisport | EU | ISO |
| --- | --- | --- | --- | --- | --- | --- | --- | --- |
| Huiyng, 2017 (Journal article;  China) | Rassegna di diritto ed Economia dello Sport | X |  | No | L | Football | Multicountry | ISO |
| Schwab, 2018 (Journal article; Australia) | International Sports Law Review Pandektis | X |  | Levels of athlete activism | Pol, L | Multisport | Multicountry | ISO |
| Wiater, 2023 (Journal article;  Germany) | Business and Human Rights Journal | X |  | No | Pol, L | Multisport | Multicountry (Russia, Belarus, Ukraine) | ISO |
| Dorskaia & Dorskii, 2021  (Journal article;  Russia) | Vestnik Sankt-Peterburgskogo Universiteta. Pravo | X |  | No | Pol, L | Multisport | Russia | ISO, NSO |
| Panagiotopoulos & Kallimani, 2017 (Journal article;  Greece) | Rassegna di diritto ed Economia dello Sport | X |  | No | Pol, L | Football | Greece | ISO, NSO |

| Scheerder, 2020 (Book chapter;  Belgium) | Routledge | X |  | Principal-agent theory | Pol, L, F, Pyr | Multisport | Multicountry | ISO |
| --- | --- | --- | --- | --- | --- | --- | --- | --- |
| Donnelly et al., 2022 (Journal article;  Canada) | International Sports Law Journal | X |  | No | Pol, L | Multisport | Canada | NSO |
| Scheerder et al., 2017 (Book chapter; Belgium) | Springer Nature | X |  | Principal-agent theory | Pol, L, F, Pyr | Multisport | Multicountry | ISO, NSO |
| García, 2024 (Journal article; UK) | International Sports Law Journal | X |  | No | Pol, L | Multisport | EU | ISO, NSO |
| García & Weatherill, 2012  (Journal article; UK) | Journal of European Public Policy |  | Interviews and document analysis | No | Pol, L | Multisport | EU | ISO, NSO |
| Kornbeck, 2022 (Book; Germany) | Taylor and Francis | X |  | No | L | Multisport | Multicountry | ISO |

| Halleux, 2015 (Report;  Belgium) | European Parliamentary Research Service | X |  | No | Pol, L | Multisport | EU | ISO |
| --- | --- | --- | --- | --- | --- | --- | --- | --- |
| Weatherill, 2012 (Book; UK) | Springer Nature | X |  | No | Pol, L | Multisport | EU | ISO, NSO |
| Kruessmann, 2019 (Journal article; Estonia) | International Sports Law Journal | X |  | No | Pol, L | Multisport | Multicountry | ISO, NSO |
| N/A, 2009 (Report; Switzerland) | International Olympic Committee | X |  | No | Pol, L, F, Pyr | Multisport | Multicountry | ISO, NSO |
| Porat, 2012  (Journal article; Israel) | Journal of European Public Policy | X |  | No | Pol | Football | Israel | NSO |
| Ioannidis, 2019 (Journal article; UK) | International Sports Law Journal |  | Interviews and surveys | No | Pol, L | Football | Multicountry (UK, Germany, France, Italy, Spain, Greece) | ISO |
| Scelles, 2017 (Book chapter; UK) | Springer Nature | X |  | The typologies established by Houlihan (1997) and Henry (2009) | Pol, L, F | Multisport | France | NSO |

| McCutcheon, 2002 (Journal article;  Ireland) | European Sport Management Quarterly | X |  | No | Pol, L | Multisport | EU | ISO |
| --- | --- | --- | --- | --- | --- | --- | --- | --- |
| García, 2016 (Journal article; UK) | Entertainment and Sports Law Journal | X |  | Agenda-setting | Pol, L | Multisport | EU | ISO |
| Lenskyj, 2018 (Book; Canada) | Emarald Publishing | X |  | No | Pol, L | Multisport | Multicountry | ISO, NSO |
| Breuer & Nowy, 2017 (Book chapter; Germany) | Springer Nature | X |  | The typologies established by Houlihan (1997) and Henry (2009) | Pol, L, F | Multisport | Germany | NSO |
| Foster, 2019  (Journal article; UK) | Entertainment and Sports Law Journal | X |  | No | L | Multisport | Multicountry | ISO, NSO |
| Forster, 2006 (Journal article; Australia) | Corporate Governance | X |  | Hirschman’s concepts of voice, exit and loyalty | Pol, L, F, Pyr | Multisport | Multicountry | ISO |
| Geeraert et al., 2014 (Journal article; Belgium) | International Journal of Sport Policy and Politics |  | Surveys and document analysis | No | Pol, L, F, Pyr | Multisport | Multicountry | ISO |

| Johnson, 1982 (Journal article; USA) | Journal of Sport & Social Issues | X |  | Easton’s concepts to domestic sports policy | Pol | Multisport | Multicountry | NSO |
| --- | --- | --- | --- | --- | --- | --- | --- | --- |
| Dolbysheva, 2022 (Journal article;  Ukraine) | Sport i Turystyka | X |  | No | Pol | Multisport | Multicountry | ISO |
| Hylton, 2017 (Journal article;  USA) | Maryland Journal of International Law | X |  | No | Pol, L | Football | Multicountry | ISO |
| Di Marco, 2022 (Journal article; France) | Netherlands Quarterly of Human Rights | X |  | No | Pol, L | Multisport | Multicountry | NSO |
| Waters, 2023  (Journal article; USA) | International Journal of the History of Sport | X |  | No | Pol, L | Multisport | Yugoslavia | ISO, NSO |
| Abrutyn, 2018 (Journal article; Canada) | Sociological Quarterly |  | Qualitative Documental Analysis | Weberian Theory, Luhmann's Social Systems Theory | Pol | Baseball | Multicountry | ISO professional sport organizations (Major League Baseball) |
| Agafonova, 2019 (Journal article;  Switzerland) | International Sports Law Journal | X |  | No | Pol, L, F, Pyr | Multisport | Multicountry | ISO |

| Foster,  2012 (Book chapter; UK) | Springer Nature | X |  | A model of internationalised and globalised sport as developed by Houlihan | L | Multisport | Multicountry | ISO |
| --- | --- | --- | --- | --- | --- | --- | --- | --- |
| Parrish, 2022 (Journal article; UK) | European Sport Management Quarterly | X |  | No | Pol, L | Multisport | EU | ISO |
| Carlsson & Lindfelt, 2010 (Journal article;  Sweden) | Sport in Society | X |  | Phenomenological analysis | Pol, L, F, Pyr | Multisport | Multicountry (Nordic countries) | NSO |
| Calo et al.,  2023 (Journal article;  Qatar) | Administrative Theory and Praxis | X |  | No | Pol, L | Multisport | Qatar | NSO |
| Minikin, 2015  (Journal article;  UK) | International Journal of the History of Sport |  | Documents and interviews | No | Pol | Multisport | Multicountry | ISO, NSO |
| Parrish, 2012  (Journal article;  UK) | European Law Review | X |  | No | L | Multisport | EU | ISO |

| Vieweg,  2014 (Journal article;  Germany) | International Sports Law Review Pandektis | X |  | No | L | Multisport | Multicountry | ISO |
| --- | --- | --- | --- | --- | --- | --- | --- | --- |
| Serra, 2020 (Journal article;  Italy) | International Sports Law Review Pandektis | X |  | No | L | Multisport | Multicountry | ISO |
| Shevchenko et al., 2016 (Journal article;  Russia) | International Sports Law Review Pandektis | X |  | No | L | Multisport | Multicountry | ISO |
| Mravec, 2021 (Journal article;  Czech Republic) | Studia Sportiva | X |  | No | L | Multisport | Qatar | ISO |
| Geeraert, 2014  (Journal article;  Belgium) | International Journal of the History of Sport |  | Documents and interviews | No | Pol, L | Football | EU | ISO, NSO |
| Zeimers et al., 2020  (Journal article;  Australia) | European Law Review |  | Survey and interviews | The professionalisation model of Nagel et al. (2015) and frameworks by Maon et al. (2010) and Baumann-Pauly et al. (2013) | F | Multisport | Belgium | ISO, NSO |

| Yaghi & Almutawwa,  2023 (Journal article;  United Arab Emirates) | Public Organization Review |  | Survey | Institutional theory | Pol, F | Multisport | United Arab  Emirates | ISO, NSO |
| --- | --- | --- | --- | --- | --- | --- | --- | --- |
| Winand et al., 2020 (Journal article;  Luxembourg) | Journal of Global Sport Management |  | Interviews and document analysis | Institutional theory, resource dependence theory and stakeholder theory | Pol, L, F, Pyr | Multisport | Scotland | NSO |
| Szwedo, 2011 (Journal article; Poland) | University of Denver Sports & Entertainment Law Journal | X |  | No | Pol, L | Football | Poland | ISO, NSO |
| Abanazir, 2022 (Book;  Turkey) | Routledge | X |  | Geerart's dimentions of sport autonomy | Pol, L, F, Pyr | Multisport | Qatar | ISO |
| Weatherill, 2017  (Book;  UK) | Oxford University Press | X |  | No | Pol, L | Football | EU | ISO, NSO |
| Caiger & Gardiner, 2001  (Book;  UK) | T.M.C. Asser Press | X |  | No | Pol, L, F, Pyr | Multisport | EU | ISO, NSO |

| Panagiotopoulos et al.,  2010 (Journal article;  Greece) | International Sports Law Review Pandektis | X |  | No | Pol, L | Multisport | EU | ISO, NSO |
| --- | --- | --- | --- | --- | --- | --- | --- | --- |
| García et al., 2023 (Journal article;  UK) | Journal of Southern African Studies |  | Interviews and document analysis | The basic concepts of policy transfer | Pol, L, F, Pyr | Multisport | Botswana | NSO |
| Sárközy, 2001 (Journal article;  Hungary) | Acta juridica hungarica | X |  | No | Pol, L | Multisport | Hungary | NSO |
| Li et al., 2001 (Book chapter; USA) | Sport Management Library | X |  | No | Pol, F | Multisport | Multicountry | ISO |
| Fischer et al., 2023 (Journal article;  Germany) | International Sports Law Journal | X |  | Concept of ‘responsible sport’ by Rook et al. | Pol, L | Multisport | Multicountry (France and Germany) | NSO |
| Rook et al., 2023  (Journal article;  Switzerland) | International Sports Law Journal | X |  | No | Pol, L | Multisport | Multicountry | ISO |
| Weatherill, 2022 (Journal article; UK) | Cambridge Yearbook of European Legal Studies | X |  | No | L | Football | EU | ISO |

| Zakharova & Melnik,  2020 (Journal article;  Russia) | Comparative Law Review | X |  | No | L | Multisport | Multicountry | ISO, NSO |
| --- | --- | --- | --- | --- | --- | --- | --- | --- |
| Budevici-Puiu et al., 2020 (Journal article;  Moldova) | Revista Romaneasca pentru Educatie Multidimensionala |  | Sociological questionnaire and observational methods | No | Pol, L, F, Pyr | Multisport | Moldova | NSO |
| Meeuwsen & Kreft, 2023 (Journal article;  Netherlands) | Sport, Ethics & Philosophy | X |  | Aristotle's ethics and contemporary political philosophy | Pol, L | Multisport | Multicountry | ISO |
| Taylor, 1988 (Journal article; UK) | International Journal | X |  | Functionalism and the state system in international relations | Pol, L | Multisport | Multicountry | ISO |
| Hoye, 2013 (Book chapter;  Australia) | Routledge | X |  | No | Pol, L, Pyr | Multisport | Multicountry | ISO |
| García, 2009  (Journal article; UK) | International Journal of Sport Policy and Politics | X |  | No | Pol, L, Pyr | Multisport | EU | ISO |
| Jedlicka, 2018 (Journal article;  USA) | International Journal of Sport Policy and Politics | X |  | International relations theory | Pol | Multisport | Multicountry | ISO |

| Allison & Monnington,  2002 (Journal article;  UK) | Government and Opposition | X |  | No | Pol | Multisport | Multicountry | ISO, NSO |
| --- | --- | --- | --- | --- | --- | --- | --- | --- |
| Lewis & Taylor, 2021 (Book; UK) | Bloomsbury | X |  | No | Pol, L, F, Pyr | Multisport | Moldova | ISO, NSO |
| Serby, 2017 (Journal article; UK) | Entertainment and Sports Law Journal | X |  | No | Pol, L | Multisport | Multicountry | ISO |
| Di Marco, 2021 (Journal article; France) | European Business Law Review | X |  | No | Pol, L, F, Pyr | Multisport | Multicountry | ISO |
| Bruyninckx, 2012 (Book chapter; Belgium) | Routledge | X |  | No | Pol | Multisport | Multicountry | ISO |
| Papaloukas, 2013  (Journal article; Greece) | International Sports Law Review Pandektis | X |  | No | Pol, L, F, Pyr | Multisport | Multicountry | ISO, NSO |
| Jevtić, 2019 (Journal article;  USA) | Physical Culture | X |  | No | Pol, L, F, Pyr | Multisport | Serbia | NSO |

| Greitens,  2021 (Book chapter;  Germany) | Springer Nature | X |  | No | Pol, L | Multisport | EU | ISO |
| --- | --- | --- | --- | --- | --- | --- | --- | --- |
| Zintz & Gérard, 2019 (Book chapter;  Belgium) | Edward Elgar Publishing |  | Surveys | No | Pol, L, F, Pyr | Multisport | Multicountry (Belgium,  Germany, Lithuania, Luxembourg,  Portugal, Slovenia, and  Turkey) | NSO |
| Coleman, 2020 (Journal article; USA) | AJIL Unbound | X |  | No | L | Multisport | Multicountry | ISO |
| García & Meier, 2022 (Journal article; UK) | Frontiers in Sports and Active Living |  | Document analyses and expert semi-structured interviews | Governance transplants | Pol, L, F, Pyr | Multisport | Multicountry (Botswana, Guatemala, Sri Lanka) | ISO, NSO |
| Budevici-puiu & Manolachi, 2022 (Journal article;  Moldova) | Revista Romaneasca pentru Educatie Multidimensionala |  | Synthesis of normative acts in the field, interview and systematic observation method. | No | Pol, L | Multisport | Moldova | ISO, NSO |
| Schimank, 2005  (Journal article;  Germany) | European Journal for Sport and Society | X |  | Sociological theories of societal differentiation | Pol, L, F | Multisport | Multicountry | ISO, NSO |

| Chappelet,  2018 (Book chapter;  Switzerland) | Edward Elgar Publishing | X |  | No | Pol, L, F, Pyr | Multisport | EU | ISO |
| --- | --- | --- | --- | --- | --- | --- | --- | --- |
| Thing & Ottesen, 2010 (Journal article  Denmark) | Edward Elgar Publishing | X |  | Foucauldian and Eliasian thinking | Pol | Multisport | Denmark | NSO |
| Flanagan, 2018 (Journal article; UK) | International Sports Law Journal | X |  | No | Pol, L | Football | Multicountry | ISO |
| Serby, 2015 (Journal article; UK) | International Sports Law Journal | X |  | No | Pol, L | Multisport | Multicountry | ISO, NSO |
| González, 2022 (Journal article; Spain) | International Sports Law Journal | X |  | No | Pol, L | Multisport | Multicountry | ISO |
| García, 2010  (Book chapter;  UK) | Routledge | X |  | Sociological theories of societal differentiation | Pol, L, Pyr | Multisport | EU | ISO, NSO |
| Baddeley, 2020 (Journal article;  Switzerland) | International Sports Law Journal | X |  | No | Pol, L | Multisport | Switzerland | ISO |

| Kalashyan,  2022 (Journal article;  USA) | European Competition Journal | X |  | No | Pol, L, F, Pyr | Multisport | Multicountry | ISO |
| --- | --- | --- | --- | --- | --- | --- | --- | --- |
| Lewandowski, 2020 (Journal article;  Poland) | Tilburg Law Review | X |  | No | Pol | Multisport | EU | NSO |
| Di Marco, 2019 (Journal article;  France) | International Sports Law Journal | X |  | Theories of corporate democracy | Pol, L | Multisport | Multicountry | ISO, NSO |
| Foster, 2007 (Book chapter; UK) | International Sports Law Journal | X |  | No | L | Multisport | Multicountry | ISO |
| Vieweg, 2000 (Book chapter;  Germany) | Springer Nature | X |  | No | L | Multisport | EU | ISO |
| Geeraert, 2019  (Journal article;  Belgium) | European Sport Management Quarterly |  | Interviews and document analysis | Compliance theory | Pol, L, Pyr | Multisport | Multicountry | ISO, NSO |
| Næss, 2022 (Book;  Norway) | Palgrave macmillan | X |  | No | Pol, L, F, Pyr | Multisport | Multicountry | ISO |

| Næss, 2020 (Journal article; Norway) | Sport, Ethics & Philosophy | X |  | No | Pol, L | Multisport (football and motorsport) | Multicountry | ISO |
| --- | --- | --- | --- | --- | --- | --- | --- | --- |
| Girginov, 2023 (Journal article; UK) | European Sport Management Quarterly | X |  | No | Pol, Pyr | Multisport | Multicountry | ISO, NSO |
| Hessert, 2021 (Journal article; Switzerland) | International Sports Law Journal | X |  | No | L | Multisport | Multicountry | ISO |
| Serby, 2016 (Book chapter; UK) | International Sports Law Journal | X |  | No | L | Football | EU | ISO |
| Modi, 2023 (Journal article;  India) | International Sports Law Journal | X |  | No | Pol, L | Multisport | Multicountry | ISO |
| Foster, 2010  (Journal article; UK) | International Sports Law Review Pandektis | X |  | No | L | Multisport | Multicountry | ISO, NSO |
| Duval, 2022 (Book chapter;  Netherlands) | Oxford University Press | X |  | No | Pol, L | Multisport | Multicountry | ISO, NSO |

| Shinohara, 2022 (Journal article; Switzerland) | International Sports Law Journal | X |  | No | L | Multisport | Multicountry | ISO, NSO |
| --- | --- | --- | --- | --- | --- | --- | --- | --- |
| Cornu et al., 2017 (Book; Switzerland) | Council of Europe | X |  | No | Pol, L, F, Pyr | Multisport | Multicountry | ISO, NSO |
| Chappelet, 2010 (Journal article; Switzerland) | Council of Europe | X |  | No | Pol, L, F, Pyr | Multisport | EU | ISO, NSO |
| Choi, 2023 (Thesis;  South Korea) | University of Ottawa |  | Documentary analysis, case studies, and semi-structured interviews | No | F | Multisport | South Korea | NSO |
| Wenn, 2024 (Journal article; Canada) | The Olympic Studies Centre | X |  | No | Pol, L, F, Pyr | Multisport | Multicountry | ISO, NSO |
| Wickstrøm & Alvad, 2017  (Report; Denmark) | Play the Game/Danish Institute for Sports Studies |  | Descriptive survey | No | Pol, Pyr | Multisport | Multicountry | NSO |
| Geeraert, 2018 (Report;  Belgium) | Play the Game/Danish Institute for Sports Studies |  | Questionnaire, interviews, and documents | No | Pol, L, F | Multisport | Multicountry (Cyprus, Denmark, Belgium (Flanders), Germany, the Netherlands, Norway, Poland, Romania, Brazil, and Montenegro) | NSO |
| Colucci & Geeraert, 2013 (Report; Italy) | Play the Game/Danish Institute for Sports Studies | X |  | No | Pol, L, F, Pyr | Football | EU | ISO, NSO |
| Mrkonjic, 2013 (Report; Switzerland | Play the Game/Danish Institute for Sports Studies | X |  | No | Pol, L, F | Multisport | Switzerland | ISO, NSO |

|  | *Note*. *Theoretical frameworks refer to a structural representation of relationships between concepts (Doherty, 2013).  ISO – International Sport Organisation, NSO – National Sport Organisation. |  |
| --- | --- | --- |
